# Quantifying the relative importance of disease-suppressive mechanisms in species mixtures: a case study of late blight in strip-intercropped potato

**DOI:** 10.1093/jxb/erag097

**Published:** 2026-02-24

**Authors:** Matthew Brandon, Zohralyn Homulle, Jacob C Douma

**Affiliations:** Centre for Crop System Analysis, Wageningen University, Wageningen 6700 AK, The Netherlands; Centre for Crop System Analysis, Wageningen University, Wageningen 6700 AK, The Netherlands; Centre for Crop System Analysis, Wageningen University, Wageningen 6700 AK, The Netherlands; University of Ghent, Belgium

**Keywords:** Disease-suppressive mechanisms, dispersal, epidemiology, microclimate, *Phytophthora infestans*, simulation model, *Solanum tuberosum*, strip intercropping

## Abstract

Numerous studies have reported disease suppression in intercropping systems, attributing it to mechanisms such as host dilution, microclimate modification, barrier effect, and induced resistance. However, the relative contributions of mechanisms to altered disease dynamics remain unclear. We combined field experiments and mechanistic modeling to quantify the importance of these mechanisms in suppressing *Phytophthora infestans* in potato intercropped with faba bean, ryegrass, or maize. Field data were used to estimate effects of disease-suppressive mechanisms on various disease processes. These were integrated into a dynamic microclimate-dependent epidemiological simulation model of late blight to predict the progression of disease severity, and the individual contribution of mechanisms. Even small differences (1–3%) in relative humidity accumulated to significantly impact disease severity. The model most accurately predicted disease suppression only when host dilution, microclimate modification, and barrier effect were combined, suggesting that each contributes substantially. Individual mechanisms varied in strength across companion crops and sometimes counteracted each other (particularly microclimate modification and barrier effect), but their combined effects consistently reduced disease. This study provides a novel framework to disentangle and quantify the contribution of disease-suppressive mechanisms in intercropping systems, enhancing our understanding of disease suppression in species mixtures, to help design cropping systems less reliant on chemical protection.

## Teaser

We propose a novel framework for disentangling the mechanisms driving disease suppression in strip-intercropping systems by integrating mechanistic effects estimated from field data into a microclimate-dependent epidemiological simulation model.

## Introduction

Modern agriculture hinges on the intensive use of pesticides, but there is a demand for more natural pest control in the growing push to transition towards agriculture with a lower impact on the environment (Tittonell, 2014; Kremen, 2020). Crop diversification at the field and landscape levels offers alternative strategies for pest and disease management (van der Werf and Bianchi, 2022). Intercropping, the simultaneous cultivation of two or more crops in the same field for at least part of their growing season (Stomph *et al*., 2020), is recognized for its potential to improve crop yield (Li *et al*., 2020), enhance crop stability (Raseduzzaman and Jensen, 2017), improve resource use efficiency (Glaze-Corcoran *et al*., 2020), and reduce disease pressure (Boudreau, 2013). Notably, disease is suppressed across numerous unique intercrop–disease combinations (Boudreau, 2013; Stomph *et al*., 2020).

Despite its promising potential, disease reduction in intercropping varies widely across studies. A meta-analysis of cereals mixed with faba bean estimated an average disease reduction of 33%, but this ranged from 20% (±9%) to 51% (±21%) depending on the crop and disease (Zhang *et al*., 2019). Additional meta-analyses have reported positive but variable pest and disease control across a range of intercrop–disease combinations (Beillouin *et al*., 2021; Chadfield *et al*., 2022), although it remains unclear to what extent the focal crop, companion, and/or the disease affects the level of disease suppression that arises from intercropping.

Disease suppression in intercrops has been attributed to several mechanisms that are linked to the introduction of a companion species. These mechanisms include an altered microclimate in the host canopy (i.e. microclimate modification; Boudreau, 1993; Gómez-Rodríguez *et al*., 2003; Schoeny *et al*., 2010; Guo *et al*., 2021), reduced density of the host crop (i.e. host dilution; Finckh *et al*., 2000; Boudreau, 2013; Zhang *et al*., 2019), the companion species acting as a barrier for the dispersal of disease propagules (i.e. the barrier effect; Gómez-Rodríguez *et al*., 2003; Schoeny *et al*., 2010; Gao *et al*., 2021), and companions influencing host plant resistance, through biotic or abiotic interactions (hereafter referred to as induced resistance; Finckh *et al*., 2000; Gómez-Rodríguez *et al*., 2003). Host dilution is frequently proposed to be the most important mechanism for disease suppression (Finckh *et al*., 2000; Boudreau, 2013; Zhang *et al*., 2019). Reducing the density of the susceptible host (by replacing it with the companion) leads to a reduction in the proportional area of susceptible hosts in a field, and an increase in the distance the inoculum must travel to infect new hosts (Burdon and Chilvers, 1982). In our study, we use the term ‘host dilution’ to refer specifically to these spatial effects of host dilution on disease dispersal. This should not to be confused with the term ‘dilution effect’, which refers to the phenomenon of reduction of disease risk with increased biodiversity (Keesing and Ostfeld, 2021).

Introducing a companion crop activates multiple mechanisms that operate simultaneously to suppress disease. For example, in a pea–cereal intercrop, reduction in *Ascochyta* blight was explained by host dilution, lowered relative humidity, and reduced splash dispersal (Schoeny *et al*., 2010). Similarly, in a pepper–maize intercrop, the density of anthracnose spores was decreased, along with increased relative humidity, and reduced temperature and sunlight intensity (Gao *et al*., 2021). These factors were found to be significantly associated with anthracnose disease incidence. In another study, the suppression of tomato early blight in tomato intercropped with marigold or pigweed was attributed to both companions acting as a barrier for spore dispersal and reducing relative humidity (Gómez-Rodríguez *et al*., 2003). Additionally, marigold exhibited allelopathic effects that inhibited *in vitro* spore germination, a response not observed with pigweed.

While these studies shed light on the complex interplay of mechanisms leading to disease suppression in intercrops, they also emphasize the challenges in assessing the contribution of individual mechanisms to overall disease suppression. Disentangling these mechanisms in field experiments can be difficult, as the mechanisms may behave differently depending on the introduced companion crop, they may interact synergistically or antagonistically, and it is not possible to isolate individual mechanisms in the field. As such, most studies fall short of identifying the relative importance of each factor in disease suppression, as they often focus on a single variable or, when multiple factors are assessed, only demonstrate correlations without partitioning contributions of various mechanisms to overall disease reductions. Process-based modeling combined with scenario analysis can help disentangle mechanisms and quantify their importance. Process-based models provide a framework for simulating systems, built from a theoretical understanding of ecological processes. Scenarios can be simulated to explore system behavior under hypothetical conditions and to analyze the divergence in outcomes as a consequence of alternative hypotheses.

Although models have been developed to explore mechanisms of disease suppression in intercropping, most past work has focused on cultivar mixtures. Process-based, epidemiological models have been used to quantify the effect of the proportion of resistant cultivars in mixtures on disease severity (Mundt and Brophy, 1988; Skelsey *et al*., 2005; Sapoukhina *et al*., 2010; Gigot *et al*., 2014; Mikaberidze *et al*., 2015), and of the proportion of the companion in species mixtures (Levionnois *et al*., 2023). The effect of the spatial arrangement has been explored by applying scenarios with explicit spatial structure, such as intra-row, random, alternate row, strip, or block mixtures (Skelsey *et al*., 2005; Gigot *et al*., 2014; Allen-Perkins and Estrada, 2019; Levionnois *et al*., 2023). Models have also assessed how plant architecture affects disease dispersal and contributes towards the barrier effect (Vidal *et al*., 2018; Levionnois *et al*., 2023) and how induced resistance is generated through cross-protection in cultivar mixtures (Lannou *et al*., 1995; Clin *et al*., 2021). Finally, work has explored the effect of management decisions, such as sowing date and fertilization, on disease progress (Levionnois *et al*., 2023). Beyond this, studies that investigate multiple mechanisms or factors simultaneously remain scare (Skelsey *et al*., 2005; Levionnois *et al*., 2023). Furthermore, to our knowledge, no modeling framework exists to quantify the effect of microclimate modification in intercropping, especially in relation to its interaction with other important disease-suppressive mechanisms.

In this study, we parameterize a process-based epidemiological model using an extensive dataset from a strip-intercrop field experiment. Strip-intercropping, hereafter referred to as strip cropping, is a form of intercropping where species are alternated in multi-row strips. We analyzed field data on late blight (*Phytopthora infestans*) in potato (*Solanum tuberosum)*, which was strip-cropped with either faba bean (*Vicia faba*), English ryegrass (*Lolium perenne*), or maize (*Zea mays*). The dependence of potato late blight on relative humidity and temperature has been well described in process-based epidemiological models (Bruhn and Fry, 1981; Andrade-Piedra *et al*., 2005a, b; Skelsey *et al*., 2009; Hjelkrem *et al*., 2021), making late blight in strip-cropped potato particularly useful as a model pathosystem for studying how intercropping influences canopy microclimate and disease development. The three companion crops, faba bean, maize, and ryegrass, have distinct characteristics, such as contrasting stature, and hence are expected to influence disease dynamics through different mechanisms. Utilizing the parameterized epidemiological model, we simulate scenarios in which single or multiple mechanisms are operating and we quantify the relative importance of individual disease-suppressive mechanisms to overall disease suppression in potato-based strip crops.

## Materials and methods

### Theoretical framework

We developed a modeling framework to quantify the relative contributions of different disease-suppressive mechanisms associated with introducing companion species through intercropping. Our study is based on data from a field experiment that investigated late blight epidemic dynamics in potato grown under four treatments: as a monoculture or as a strip crop (a form of intercropping) with either faba bean, maize, or ryegrass (Homulle *et al*., 2024). The field experiment measured disease severity, canopy temperature and relative humidity, aerial particle deposition, wind speed, and plant resistance. These measurements may serve as indicators of mechanisms relevant to potato-based strip-cropping systems, namely microclimate modification, host dilution, barrier effect, and induced resistance.

A process-based late blight epidemic model (Skelsey *et al*., 2009) was used to simulate disease dynamics from canopy microclimate (temperature and relative humidity) in response to microclimate modification by the companion crops. For the three remaining mechanisms, we estimated rate modifiers to represent the mechanism-specific effect that a companion crop has on a disease process. Rate modifiers determine how strongly individual disease-suppressive mechanisms modify the rate of disease processes in strip crops relative to the monoculture. Statistical models were used to estimate rate modifiers for each companion for (i) the barrier effect on spore deposition, using particle deposition measurements and (ii) for induced resistance on infection rate using detached leaf resistance measurements. A dispersal kernel model was used to estimate a rate modifier for the effect of host dilution on spore deposition in the monoculture versus the strip crops. Together with strip-crop microclimate data, these rate modifiers were used to adjust epidemic model parameters to simulate the effects of strip cropping on disease severity overall.

**Fig. 1.**
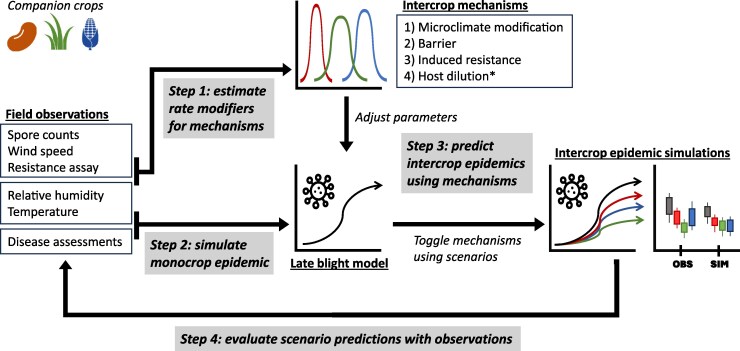
Conceptual diagram of the modeling approach. This diagram outlines the methodology used to model the effect of disease-suppressive mechanisms in strip crops on late blight epidemics in potato, which consists of four steps. Step 1: field observations are used to estimate rate modifiers which estimate the strength of disease-suppressive mechanisms in strip crops relative to the monoculture. Rate modifiers are used (in Step 3) to change the rate parameters in the late blight model. Step 2: a general late blight epidemic model is parametrized to fit observed disease progress in the monoculture. Step 3: disease progress in strip-crop treatments is predicted by inputting companion-specific strip-crop canopy microclimate data and adjusting model parameters according to the rate modifiers estimated in Step 1; mechanisms are toggled ON/OFF in a full factorial design. Step 4: disease progress under all scenarios is compared with observed disease severity. Refer to the text for more details. *The effect of host dilution was estimated using a spore dispersal model and parameters estimated in a previous study (Paysour and Fry, 1983; Skelsey *et al*., 2005).

The approach we developed follows four steps (Fig. 1). Step 1: field measurements were analyzed to estimate rate modifiers for the effects of host dilution, barrier effect, and induced resistance. Step 2: parameters of an epidemic model were calibrated to the growing season, using microclimate measured in the monoculture to replicate disease progress observed in the monoculture. Step 3: microclimate data measured in the strip crops and the estimated rate modifiers were switched on or off in the epidemic model in various combinations to simulate disease progress, exploring the effect of mechanisms in isolation and combined on disease progress. Step 4: simulated disease severity for the different scenarios were compared with severity observed in the strip-crop treatments to infer the importance of the mechanisms. A conceptual diagram of the approach described is shown in Fig. 1. Before we describe the four steps in more detail, we first describe the field set-up and experimental measurements, followed by a description of the process-based epidemic model of late blight.

### Field set-up and experimental measurements

The main data used in our approach were collected in a field experiment conducted at the organic experimental farm of Wageningen University, the Netherlands (51.99°N, 5.65°E), between April and August 2022 (Homulle *et al*., 2024). The experiment included four treatments with four replications each, divided across two locations ∼800 m apart: (i) a monoculture potato treatment (‘mono’), and (ii) potato–faba bean (‘bean’), (iii) potato–ryegrass (‘grass’), and (iv) potato–maize (‘maize’) strip-crop treatments. Each plot measured 21 m×24 m. A replacement design was used, with strip-crop plots divided into seven strips of equal 3 m width, alternating over four companion strips and three potato strips. In all plots, potato was planted at a row distance of 75 cm and an intra-row distance of 40 cm. This resulted in four-row strips of potato in the strip-crop plots. Potato (cv. Agria) was planted on 17 May 2022. Agria is moderately resistant to foliar late blight. In the respective plots, faba bean (cv. Cartouche) was planted on 3 May in six-row strips, while maize (a mixture of 73% Autens KWS and 27% LG30.179) was planted on 29 April in four-row strips. English ryegrass (*Lolium perenne*) was also sown on 29 April, and was mowed multiple times over the season to be kept short. The fields were managed organically; organic fertilizer was used, and no pesticides or irrigation were applied. See Homulle *et al*. (2024) for detailed management information.

The summer of 2022 was warm, sunny, and dry compared with typical conditions for the area. June was warm and wet, with a mean daily temperature of 17.6 °C and 70.3 mm of cumulative rainfall. July was slightly warmer than typical, but very dry and sunny, with a mean temperature of 19.2 °C and 21.3 mm of rainfall. Extreme high temperatures reaching above 35 °C were recorded on 19 July. August was very warm and dry with a mean temperature of 21.2 °C and 9.7 mm of rainfall.

Throughout the growing season, measurements were collected in each treatment for (i) disease severity, (ii) canopy microclimate, (iii) spore dispersal, and (iv) host resistance (Table 1). (i) Disease severity was assessed for 24 potato plants in each plot seven times between the first observation of late blight symptoms on 8 July 2022, until termination of the crop on 11 August 2022; the plants to be monitored were selected with stratified random sampling. Initially, infected leaflets were counted, and later severity was visually estimated. (ii) In three plot repetitions of each treatment, canopy temperature and relative humidity were logged every 10 min from 16 June until 9 August at either the center of the monoculture plots, or at two central positions in each strip-crop plot: within the middle potato strip, and at the border row of the middle potato strip. Before June 16, local weather data were used to simulate leaf area growth in potato. (iii) To evaluate the effect of wind speed on spore dispersal, wind speed was measured at the periphery of the field every 10 min. Next, a trap equipped with three microscope slides for catching incoming particles was positioned at the center of each plot at a height of 10–20 cm above the canopy. Particle sampling occurred every few days between 29 June and 1 August, with collection running from 16.00 h until 09.00 h the following day, for 21 collection dates in total. The numbers of particles with a size similar to *P. infestans* spores (estimated with a cross-sectional area within 314–1257 mm^2^) were counted from four microscope images (20 mm^2^) per slide, for a total of 12 counts per plot per collection date. (iv) To assess *P. infestans* resistance, a detached leaf assay (DLA) was performed on 23 June, prior to the first field observation of *P. infestans*. From each plot, 14 randomly sampled potato leaflets were inoculated with 10 isolated droplets of *P. infestans* spore suspension; the number of successful infections per leaflet was counted after 5 d. For further details about the measurements refer to Homulle *et al*. (2024, 2025).

**Table 1.** List of data used for modeling late blight epidemics in monoculture and strip-crop potato systems

	Measurement	Frequency/date	Measurement source	Step(s) used
Late blight severity	No. of infected leaflets per plant	July 11–12, 14–15, 18–19, 21–22, 25–26, 29–30 August 3–4	*In situ*	2, 4
	Estimated severity (%)			
Microclimate	Relative humidity (%)	10 min	*In situ*	2, 3
		Hourly	Local weather station	
	Temperature (°C)	10 min	*In situ*	2, 3
		Hourly	Local weather station	
	Precipitation (mm h^–1^)	Hourly	Local weather data	2, 3
Spore dispersal	No. of trapped particles per slide image	Every 1–3 d	*In situ*	1
	Wind speed (m s^–1^)	10 min	*In situ*	1
Host resistance	No. of lesions per detached leaflet	June 23	*In situ*	1

There are two sources of relative humidity and temperature data; these time series were experimentally measured *in situ*, and weather station data were used to impute missing/erroneous readings and data outside of the date range of experimental measurements. The steps of the methodology in which these data were used are listed (refer to the text for details).

### Epidemic model description

**Fig. 2.**
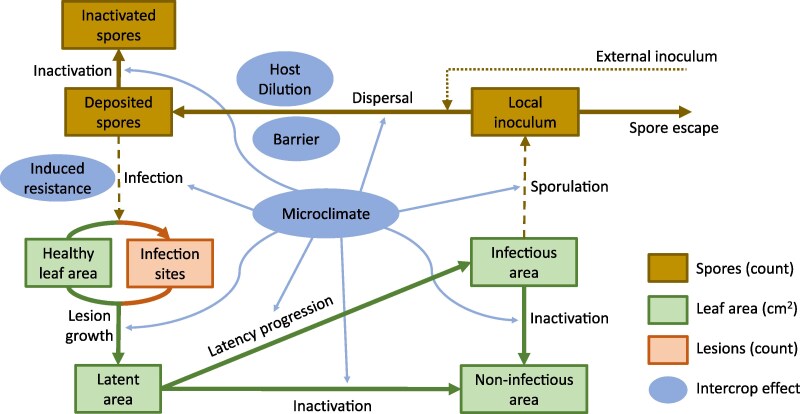
Conceptual diagram of the process-based late blight model and disease processes affected by intercropping. The model was used to predict late blight disease progress as a function of hourly relative humidity, temperature, and precipitation. Disease progress in strip crops is modeled through introducing disease-suppressive mechanisms of strip cropping. Disease-suppressive mechanisms are represented by the blue ovals and are positioned adjacent to the disease process they affect. For the effect of microclimate modification, blue arrows indicate the processes dependent on microclimate. Green boxes represent host leaf area state variables (healthy and diseased), brown boxes are spore number state variables, and the single red box represents the number of lesions, which is required to model variable latency progression. Solid green and brown arrows indicate flows within leaf area or spore number state variables, respectively. Dashed brown arrows depict the flow between leaf area and spore number state variables, either through the deposition of spores on leaves and subsequent infection, or through sporulation from leaves. The dotted brown arrow represents the initial introduction of external inoculum into the system. The host growth model components have been excluded for simplicity. Note that rainfall does not vary in response to treatment, but it is used in the estimation of leaf wetness upon which infection is dependent.

We based our epidemic model on an existing process-based model that simulates the seasonal development of late blight in a monoculture potato canopy (BLIGHTTIME; Skelsey *et al*., 2009), conceptually described in Fig. 2. The model was adapted to take hourly times series of relative humidity, temperature, and precipitation as input values to calculate rate variables for processes in the infection cycle (e.g. deposition efficiency, infection efficiency, and latency progression rate) in addition to host growth (e.g. leaf growth rate and leaf death rate). The model integrates these rate variables to produce an hourly disease severity output—calculated as the proportion of total leaf area that is diseased—over the duration of input data, starting from the date of first infection. Relative humidity and temperature were measured in each canopy (aggregated to hourly means), while precipitation data were obtained from a local weather station (Veenkampen; 51.98138°N, 5.62148°E).

Further modifications to BLIGHTTIME included: a leaf wetness infection requirement (blight hour function); a maximum lesion age; a maximum lesion number per plant; temperature-dependent latency progression; and high-temperature lesion inactivation. These functions were added to account for the high temperatures observed in 2022, which had an effect on lesion growth that could not be accounted for in the original model. The blight hour function sets a temperature-dependent leaf wetness duration requirement for infection to occur at any given hour (Rotem *et al*., 1970; Hartill *et al*., 1990; Zwankhuizen and Zadoks, 2002; Hjelkrem *et al*., 2021). The maximum lesion age inactivates lesions after 15 d (Bruhn and Fry, 1981), while the maximum lesion number sets a limit to the number of lesions which can be initiated on a host plant over a season. The temperature-dependent latency progression function modulates the rate at which lesion area progresses from latent to infectious according to temperature (Narouei-Khandan *et al*., 2020), and the high-temperature lesion inactivation function inactivates lesions as temperatures reach a high temperature threshold (Crosier, 1934; Wallin and Hoyman, 1958). For further details on these modifications, refer to Supplementary Protocol S1.

### Modeling workflow

#### Step 1: estimating the effects of companion crops on disease processes

The effects of host dilution, barrier effect, and induced resistance on disease progress are introduced by adjusting corresponding disease process rate parameters with rate modifiers, estimated from field measurements. For microclimate, we directly input treatment-specific microclimate data into the BLIGHTTIME model. Specifically, rate modifiers for host dilution and barrier effect adjust the deposition efficiency parameter (the proportion of spores landing on potato leaves), while a rate modifier for induced resistance adjusts the infection efficiency parameter (the probability that a spore successfully infects a leaf; see Fig. 2). These rate modifiers are calculated for strips crops relative to the monoculture treatment.

To illustrate how rate modifiers for disease-suppressive mechanisms have been implemented in the model, we provide an example for how infection efficiency is calculated within the model for potato grown with different companions. Infection efficiency at time *t*, for potato intercropped with crop *i* ($IE_{it}$) is calculated as:


$$IE_{it}=IE_{\max}\times f(T_{it},\;RH_{it})\times rIE_{i}$$

**Fig. 3.**
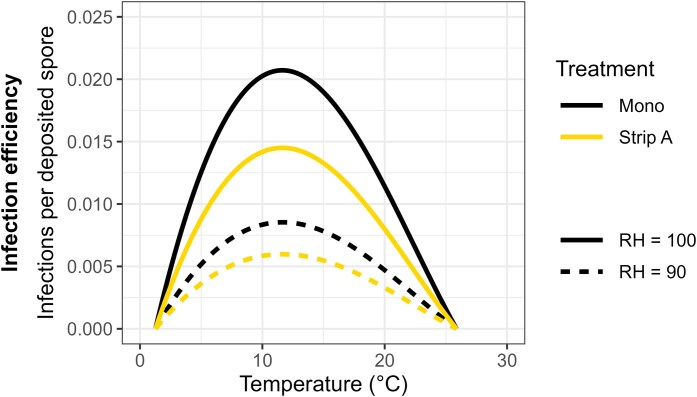
Illustrative example of how the effect of companion crops on infection efficiency is implemented in the model. The maximum infection efficiency IE_max_ for monoculture is 0.021 at ∼11.6 °C; the real infection efficiency is calculated at each time step dependent on measured hourly relative humidity (RH) and temperature. If a strip crop is simulated, this is then modified both by using the measured microclimate data specific to the strip crop and by multiplying the infection efficiency with the rate modifier representing the effect of induced resistance (as measured through a detached leaf assay). The relative infection efficiency rate modifier used for Strip A is 0.7; this is a mock value which is used in this figure as an example (the effect of strip cropping may also be disease enhancing).

$IE_{\max}$ is a model parameter for the maximum infection efficiency possible in a monoculture. $IE_{\max}$ is adjusted according to the temperature (T) and relative humidity (RH) in treatment *i* at time *t* by multiplying by $f(T_{it},\;RH_{it})$, which represents the effects of temperature and relative humidity on infection efficiency, and as such accounts for differences in microclimate induced by strip cropping with companion crop *i*. The infection efficiency can be further adjusted by multiplying by $rIE_{i}$, a parameter that accounts for the effect a companion crop *i* has on infection efficiency, for example by inducing resistance (e.g. as measured in a detached leaf assay). In all monoculture simulations, this rate modifier ($rIE_{i}$) is set to 1. A visual example is provided in Fig. 3 for how infection efficiency varies depending on temperature and relative humidity (at 100% and 90%), and mock treatment ‘Strip A’ lowers the infection efficiency compared with the monoculture, with a mock rate modifier value of 0.7.

##### Companion crop microclimate modification

Microclimate measurements (relative humidity and temperature) collected in the potato canopy of the monoculture and the three strip-crop treatments are individually input to the BLIGHTTIME model, along with precipitation data obtained from a nearby weather station. We refer to relative humidity and temperature measurements as ‘microclimate data’ because they are treatment specific, whereas precipitation from local weather data does not vary by treatment. Using canopy measurements from each treatment allowed us to predict how companion crops influences disease progression by modifying the microclimate, because microclimate affects numerous disease processes. Notably, relative humidity and temperature are used in the calculation of nearly all disease rate parameters (blue arrows in Fig. 2: infection efficiency, lesion growth rate, latency progression, sporulation efficiency, spore deposition efficiency, and lesion inactivation).

##### Estimating the barrier effect of companion crops

As an indicator of the barrier effect of companion crops, longitudinal aerial particle counts were analyzed as a response variable using a generalized linear mixed effect model. Treatment (categorical), mean daily wind speed (continuous), and their interaction were specified as fixed effect predictors, while sampling location (categorical; microscope slides nested in particle traps, one trap per plot, with plots nested in field location) and date of collection (categorical) were specified as random effects. A negative binomial distribution (NegBin) with log link was used to model the distribution of the particle counts. The maximum likelihood estimates of particle count per treatment, including their uncertainty, were used to generate probability distributions for relative spore counts ($rS_{i}$, Equation 1). Relative spore counts were then used as rate modifiers for spore deposition in BLIGHTTIME.


$$S_{ijt}∼\mathrm{NegBin}(\mu_{ijt},k_{ijt})$$

$$\log\;(S_{ijt})=\beta_{0}+\beta_{1i}+\beta_{2}W_{t}+\beta_{3i}W_{t}+\alpha_{t}+\alpha_{f}+\alpha_{\;fp}+\alpha_{\;fps}$$

$$rS_{it}=\frac{S_{it}}{S_{\mathrm{mono},t}}=\frac{\exp\;(\beta_{0}+\beta_{1i}+\beta_{2}W_{t}+\beta_{3i}W_{t})}{\exp\;(\beta_{0}+\beta_{2}W_{t})}=\exp\;(\beta_{1i}+\beta_{3i}\times W_{t})$$

(1) $$rS_{i}=\frac{\sum_{t=1}^{n}\;\exp(\beta_{1i}+\beta_{3i}\times W_{t})}{n}$$

$S_{ijt}$ is the spore count of observation *j* sampled from slide *s*, of trap *t*, of field *f*, for each collection date *t*. $\mu_{ijt}$ is the estimated mean spore count, and $k_{ijt}$ is the dispersion parameter. $\beta_{0}$ represents the constant intercept for spore counts in monoculture, and $\beta_{1i}$ are the effects of strip-crop treatments on spore counts. $W_{t}$ is the mean wind speed during spore collection intervals for each date, and $\beta_{2}$ is the marginal effect of wind speed on spore counts. $\beta_{3i}$ represents the interaction between wind speed and strip-crop treatment. $\alpha_{t},\;\alpha_{f},\;\alpha_{\;fp},\;\mathrm{and}\;\alpha_{\;fps}$ are the random effects of collection date, and nested field, trap, and slide sampling location for each observation. $rS_{it}$ represents the relative spore dispersal under strip-crop treatments, relative to the monoculture, at time *t*. $rS_{i}$ aggregates a mean relative spore dispersal which accounts for differences in wind speeds across collection dates. A distribution of $rS_{i}$ is calculated which accounts for uncertainty in $\beta_{1i}$ and $\beta_{3i}$.

##### Estimating the effect of induced resistance due to intercropping

As the indicator for induced resistance, infection counts from a detached leaf assay were analyzed as a response variable using a generalized linear mixed effect model. Treatment was the only fixed effect predictor, while sampling location (plot nested in field location) was specified as a random effect. A zero-inflated binomial distribution with logit link was used to model the distribution of infection counts. The maximum likelihood estimates of infection proportions per treatment, including their uncertainty, were used to generate probability distributions for infection rates relative to the monoculture treatment ($rIE_{i}$, Equation 2). These were used as rate modifiers for infection efficiency in BLIGHTTIME.


$$\mathrm{Icoun}t_{ij}∼\mathrm{Bin}(10,\;\pi_{ij})\cap \mathrm{Bernoulli}(\;p_{i})$$

$$\log\;\left(\frac{\pi_{ij}}{1-\pi_{ij}}\right)=\beta_{0}+\beta_{1i}+\alpha_{f}+\alpha_{\;fp}$$

(2) $$rIE_{i}=\frac{\pi_{i}}{\pi_{\mathrm{Mono}}}=\frac{\left[\frac{\exp\;(\beta_{0}+\beta_{1i})}{1+\exp\;(\beta_{0}+\beta_{1i})}\right]}{\left[\frac{\exp\;(\beta_{0})}{1+\exp\;(\beta_{0})}\right]}$$

$\mathrm{Icoun}t_{ij}\;$ is the infection count recorded for the leaflet *j* sampled from plot *p*, of field *f* for treatment *i*. $\pi_{ij}$ is the estimated proportion of inoculation sites which resulted in a successful infection modeled using a binomial distribution (Bin), and $p_{i}\;$is the probability that a zero count is observed (zero-inflation), modeled using a Bernoulli distribution. $\beta_{0}$ represents the constant intercept for infection counts in monoculture, and $\beta_{1i}$ are the effects of strip-crop treatments. $\alpha_{f}$ and $\alpha_{\;fp}\;$are nested random effects for the field and plot sampling locations of leaflets.

##### Simulating the effect of host dilution

A dispersal model was used to simulate a plot of potato plants in a monoculture versus a strip-crop arrangement and calculate the proportion of spores that land on each host plant. In the dispersal model for the strip-crop plots, potato plants were removed from sections where the host plant was replaced by a non-host companion, and the proportion of spores that land on host versus non-host plants was calculated. Thus, the reduction in spores due to strip cropping does not vary between strip-crop treatments, because the spatial arrangement of potato plants was identical regardless of the companion, while the potential (additional) barrier effect of the companion crop was treated as an independent effect. The layout of experimental plots was modeled to scale, according to the spatial arrangement of the monoculture or strip-crop plots, with a grid (21 m×24 m) containing rectangular (75 cm×40 cm) host plant grid cells and, for the strip-crop plots, non-host grid sections representing companion strips. For our estimation, we assumed that all host grid cells released the same number of spores and that their deposition distances were described by identical radial Laplace dispersal kernels (Skelsey *et al*., 2005). The steepness of the kernels was parametrized with a dispersal gradient factor, α, which for two different plots was estimated to be 0.82 m^−1^ and 1.09 m^−1^ for short-range dispersal of *P. infestans* spores (Paysour and Fry, 1983). Through integration of the dispersal kernels across all hosts plants, calculating total spores deposited relative to total spores released gave the overall probability of spores landing on host plants. The effect of host dilution was calculated as the ratio of this successful deposition probability in the strip-crop arrangement relative to the monoculture arrangement. Uncertainty in α was accounted for by using a plausible uniform distribution of ∼U(0.685,1.225) (m^−1^) which is double the range reported by Paysour and Fry (1983). This yielded a distribution of estimates for the reduction in spore deposition in strip-crop plots relative to the monoculture. These were used as the rate modifiers to adjust the BLIGHTTIME deposition efficiency parameter for strip crops. See Supplementary Protocol S2 for further details.

#### Step 2: standardization of the epidemic model with monoculture field data

Using replicate microclimate data from the monoculture, certain model input values were adjusted to calibrate the simulation of disease progression in the monoculture to match field observations of disease progress in the monoculture. Specifically, input values related to the initiation of the epidemics were set using these monoculture data. The date of inoculation was set following the procedure of Andrade-Piedra *et al*. (2005a). Inoculation load was set so that the simulated rate of disease progression in the monoculture matched the observed disease severity data. Data from the strip-crop treatments were not used in this step. These settings were maintained in all subsequent strip-crop simulations.

#### Step 3: prediction of strip intercropping disease dynamics

Using the rate modifiers that were derived in Step 1, and the microclimate data measured in the strip crops, disease progress in the strip crops was predicted. The importance of different disease-suppressive mechanisms was explored in a full factorial design in which the four mechanisms were presumed to be active or inactive, leading to 16 different scenarios. When mechanisms are switched on, rate modifiers for the mechanisms adjust the associated disease process rate parameter: host dilution and barrier effect modify the spore deposition efficiency, while the effect of induced resistance modifies the infection efficiency (see Fig. 2). Conversely, when mechanisms are switched off, the rate modifiers were set to 1, resulting in identical rate parameters to those in the monoculture.

Uncertainties in the effect of host dilution, barrier effect, and induced resistance were represented by probability distributions estimated for the corresponding rate modifiers. Their uncertainty was propagated to the modeled disease severity output. Uncertainty in microclimate modification was modeled by confidence bands produced using the hourly mean and SE of replicate relative humidity and temperature time series. A total of 500 simulations were run per treatment per scenario, with independent randomly sampled rate modifiers and microclimate confidence band contours. In scenarios with microclimate modification switched off, microclimate for the strip crops was sampled from monoculture microclimate confidence bands. Relative area under the disease progress curve was calculated for each simulation (rAUDPC_sim_) and observations (rAUDPC_obs_) using the span of observed severity dates, generating distributions which could subsequently be compared.

#### Step 4: ranked evaluation of disease-suppressive mechanisms

For each strip-crop treatment, under each scenario, a mean disease progress curve (DPC) was calculated from the DPCs produced from individual microclimate replicates. Mean absolute error (MAE) was calculated as the mean residual variance between observed severity assessments and DPCs. Scenarios were ranked within and across treatments, with the lowest MAE indicating the best prediction.

When considering uncertainty in the mechanisms, observed rAUDPC_obs_ values were compared against scenario–treatment rAUDPC_sim_ distributions. The prediction accuracy of simulated data was assessed by ranking the scenarios by predictive likelihood, which reflects the likelihood of the data being accurately described by model predictions (under each scenario). The likelihood of rAUDPC_obs_ values was obtained by using the predicted distributions in rAUDPC_sim_ as a probability distribution. Lower negative log-likelihood (NLL) indicated an improved prediction accuracy.

The BLIGHTTIME model was programmed in R, and all statistical analyses and data visualization were conducted in R (R Core Team, 2023). Generalized linear mixed models were fitted using glmmTMB (Brooks *et al*., 2017). Figures were generated using ggplot2 (Wickham, 2016), with density plots generated using ggformula (Kaplan and Pruim, 2023).

## Results

### Estimation of effects of strip intercropping on disease processes (Step 1)

#### Companion crop microclimate modification

Microclimate differed consistently between the different strip-crop treatments. Overall, relative humidity was consistently highest in potato strip-cropped with maize (‘potato–maize’; see Supplementary Fig. S1A for 24 h mean measured canopy relative humidity). During the night-time hours, the second highest relative humidity was observed in the monoculture. Mean relative humidity was generally lowest in potato strip-cropped with ryegrass (‘potato–ryegrass’), although monoculture more frequently showed low relative humidity extremes. These extremes skewed the hours spent below the 50% relative humidity threshold to a higher number in monoculture than in potato–ryegrass (Table 2). The lowest relative humidity during the night was measured in potato strip-cropped with faba bean (‘potato–faba bean’), but relative humidity in potato–faba bean remained higher than in monoculture and potato–ryegrass during the daytime. Clear patterns distinguishing mean temperature between treatments were not observed (Supplementary Fig. S1B).

**Table 2.** Mean number of hours per day in July 2022 that relative humidity falls above or below model thresholds for host–pathogen processes

Treatment	RH ≥95% (h d^–1^)	RH ≥87% (h d^–1^)	RH <60% (h d^–1^)	RH <50% (h d^–1^)
Mono	***7.2*** ±***4.4***	***11.0*** ±***4.7***	***4.5*** ±***4.8***	***2.6*** ±***4.0***
Bean	6.1±4.8	10.8±4.7	3.8±4.6	1.9±3.5
Grass	6.2±4.4	10.3±4.4	***4.7*** ±***4.6***	***2.3*** ±***3.6***
Maize	***7.8*** ±***4.7***	***11.9*** ±***4.4***	3.5±4.3	1.5±3.0

This is calculated as a mean for each treatment from relative humidity (RH) measured in monoculture (‘mono’), potato–faba bean (‘bean’), potato–ryegrass (‘grass’), and potato–maize (‘maize’) plots. RH thresholds in the epidemic model of 95% and 87% are the minimum RH required for sporulation and the minimum RH required for leaf wetness, respectively. The 60% and 50% thresholds are the minimum RH at which infection and lesion growth can still occur, respectively. Raw means are presented ±SD across replicates and days. Differences in means are not significant. The values in bold italics are the two highest means in each threshold category.

Considering the sensitivity of *P. infestans* to relative humidity, the mean number of hours per day when measured relative humidity exceeded or fell below critical BLIGHTTIME thresholds for relative humidity were calculated (Table 2). These indices further demonstrate that potato–maize, followed by monoculture, spent the most hours above the upper thresholds for relative humidity (95% for sporulation and 87% for leaf wetness). However potato–maize also spent the fewest hours below lower thresholds for relative humidity, while monoculture spent the most hours below the lower threshold of 50% relative humidity for lesion growth, and the second most hours below 60% relative humidity for infection. Potato–ryegrass and potato–faba bean spent the fewest hours above upper thresholds for relative humidity (95% and 87%); however potato–faba bean spent fewer hours below lower thresholds (60% and 50%). Potato–ryegrass spent the most hours below 60% relative humidity, and the second most hours below 50% relative humidity.

#### Effect of strip intercropping host dilution

Applying dispersal kernel models to the experimental layout of monoculture and strip-crop plots, 88% and 58.3% of released spores landed on host plants in the experimental plots, respectively (evaluated for the mean dispersal gradient, α=0.955 m^–1^. Thus, the mean proportion of spores that were deposited on hosts in strip crops is 66% of the deposition on hosts in the monoculture. Over the range of uniformly distributed α assessed, deposition relative to the monoculture was approximately uniformly distributed from 60% to 72%.

#### Effect of companion crop barrier and induced resistance

**Fig. 4.**
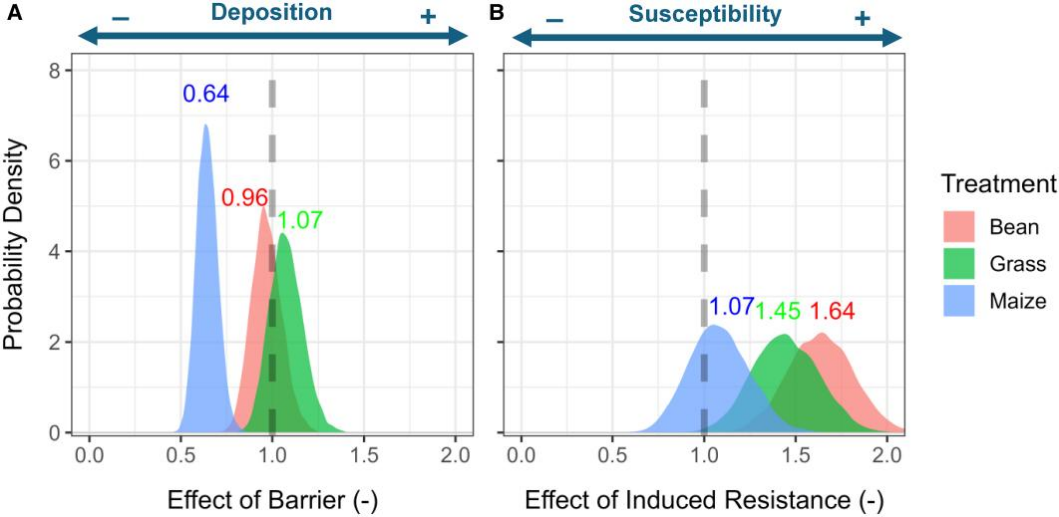
Probability density curves for estimated rate modifiers. These represent the effect of companion crops on (A) spore deposition efficiency through the barrier effect mechanism and (B) infection efficiency through the induced resistance mechanism. Effect multipliers were derived from experimental measurements and are calculated relative to the monoculture (vertical dashed gray line). The distributions are labeled with their respective means.

In assessing spore counts relative to monoculture, the treatment coefficient estimates on a log scale (estimate ±SE) were positive for potato–bean and potato–ryegrass (although insignificant), and significantly negative for potato–maize, indicating that spore deposition is reduced when potato is strip-cropped with maize (see Supplementary Table S1 for generalized linear mixed effect model coefficients). Coefficient estimates for the treatment–wind interaction term were negative for all companion crops, suggesting a reduction in deposition in strip-crop plots as wind speeds increase. Assembling the coefficient estimates using Equation 1 and propagating their uncertainty produced distributions for rate modifiers linked to the barrier effect. These indicate an increase in deposition efficiency relative to monoculture (mean ±SD) in potato–ryegrass and a decrease for potato–faba bean and potato–maize (Fig. 4A; Supplementary Table S1).

Treatment coefficient estimates on a logit scale (estimate ±SE) for detached leaf assay infection rates were positive for all companion crops. Rate modifiers (mean ±SD) of infection efficiency were assembled using Equation 2, representing the effect of induced resistance. These suggested that susceptibility to *P. infestans* was increased to varying degrees in the strip crops (Fig. 4B; Supplementary Table S1).

### Simulating monoculture epidemics (Step 2)

**Fig. 5.**
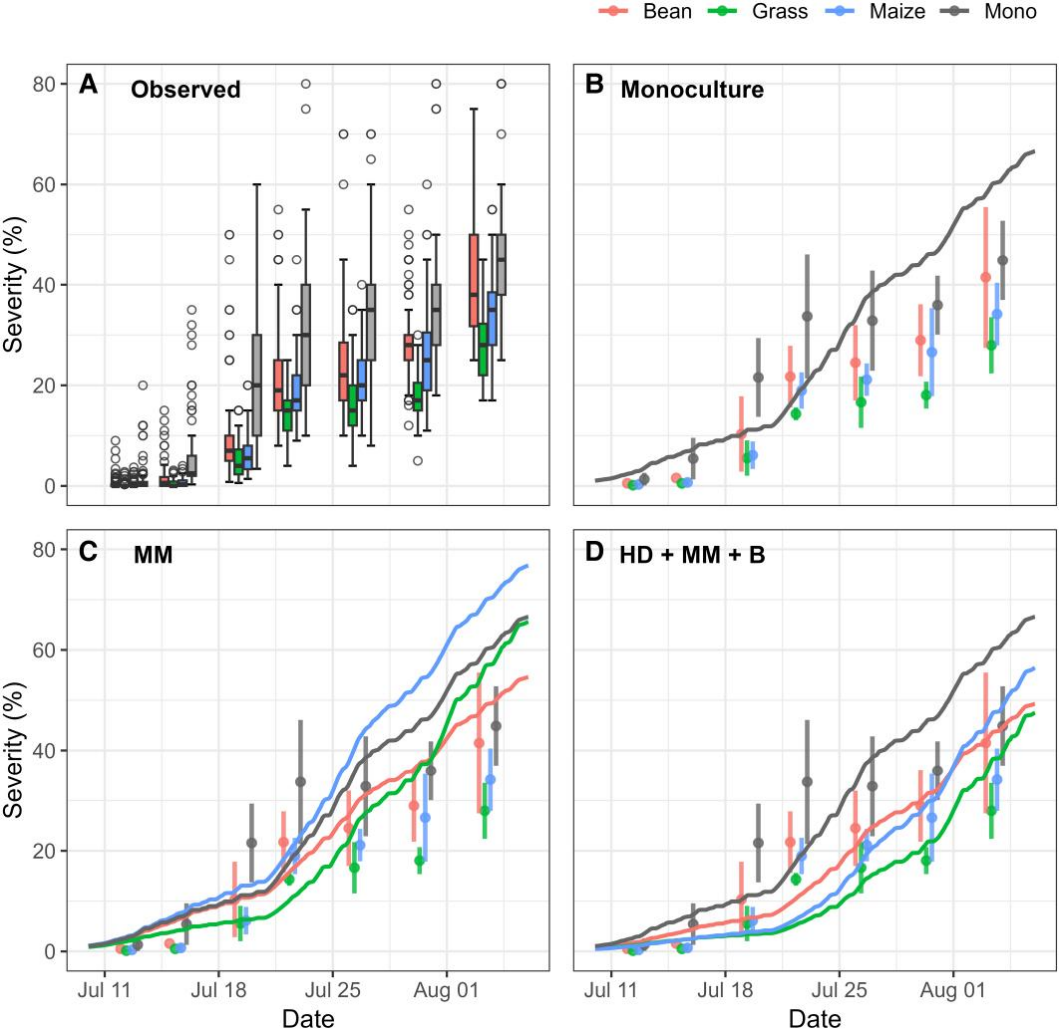
Comparison of observed disease severity and simulated disease severity in selected strip-cropping scenarios. (A) Boxplots of observed disease severity, which was assessed seven times between 11 July and 4 August 2022 for randomly selected plants from four replicate plots of each treatment; scattered points represent outliers. Lines in (B–D) represent the simulated disease progress curves (DPCs) in the monoculture (B), including the effect of measured canopy microclimate in the strip crops on disease severity (C; ‘MM’), and the simulated DPC when combining the effects of host dilution, microclimate modification, and the barrier effect (D; ‘HD+MM+B’). Points with error bars in (B–D) represent the observed mean severity ±1 SD, representing the same observed data depicted in (A). Only three of 16 scenarios are shown (refer to Supplementary Fig. S2 for figures of all scenarios). Mechanism abbreviations: HD (host dilution); MM (microclimate modification); B (barrier effect).

Date of inoculation and inoculation load were adjusted to improve the fit of simulated monoculture DPCs to disease severity data collected in the monoculture (Fig. 5A). The mean monoculture DPC (Fig. 5B; *R*^2^=0.57) was simulated with inoculation date set to July 2 (6 d prior to the first field observation of late blight symptoms). Inoculation load was set to 3500 spores per plant for three consecutive hours to initiate the epidemic, representing an incoming spore cloud that enters the field for 3 h. This inoculation load resulted in ∼22 infections per plant in the monoculture by the end of the inoculation period. The inoculation date and load settings were used in all subsequent strip-crop simulations across all scenarios (Fig. 5).

### Scenario analysis of strip intercropping disease dynamics (Steps 3 and 4)

**Fig. 6.**
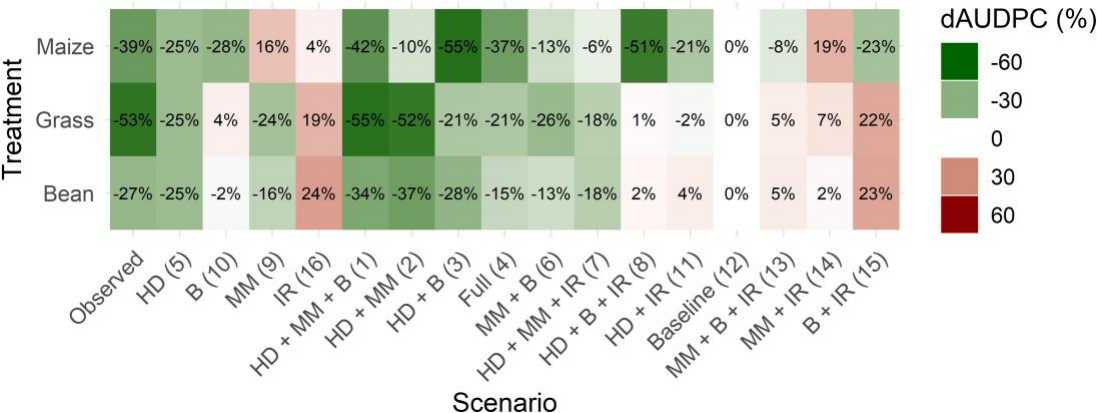
Heatmap of percentage change in rAUDPC (dAUDPC) in strip crops relative to monoculture as observed and simulated in strip-cropping scenarios. dAUDPC refers to the percentage change difference in relative area under the disease progress curve (rAUDPC). The left-most ‘Observed’ dAUDPCs were calculated from observed disease severity. This is followed by dAUDPCs calculated from disease progress curves (DPCs) simulated in scenarios of single active mechanisms (HD, B, MM, or IR), and then by those scenarios combining toggled active mechanisms. The scenarios are ranked by mean absolute error (MAE) from left (best fit) to right (worst fit); rankings are indicated in parentheses. ‘Baseline’ represents the scenario in which no disease-suppressive mechanisms are active, and strip crops are identical to the monoculture. ‘Full’ represents the scenario in which all mechanisms are active. Mechanism abbreviations: HD (host dilution); MC (microclimate modification); B (barrier effect); IR (induced resistance).

First, individual microclimate replicates were used to simulate DPCs across all 16 scenarios, consisting of a full factorial design which toggled the four mechanisms on or off (Supplementary Fig. S2). Visually, the mean DPCs of the scenario which combined host dilution, microclimate modification, and the barrier effect (Fig. 5D) most closely fitted the observed severity (Fig. 5A). This was supported by the MAE of this scenario ranking highest (Fig. 6). Host dilution was included in seven of the eight highest-ranking scenarios, microclimate modification and barrier were included in five, while only three included induced resistance. Inclusion of induced resistance resulted in a high overestimation of disease severity in potato–faba bean and potato–ryegrass. When ranking only for potato–maize, induced resistance was included in the highest-ranked scenario, which combined host dilution, barrier effect, and induced resistance. However, this required the exclusion of microclimate modification, since both of these mechanisms were predicted to promote late blight in potato–maize. Microclimate modification had a disease-suppressive effect in potato–ryegrass and in potato–faba bean, although there was higher variation in potato–faba bean (Fig. 5C); this suppression was driven by reduced relative humidity compared with the monoculture, in particular during the night-time. Conversely, microclimate modification in potato–maize promoted disease due to elevated relative humidity compared with the monoculture. Interestingly, the effect of different mechanisms on disease severity were of comparable magnitude for certain companion crops. For example, host dilution leads to a reduction in disease severity of 25% in all strip crops, the barrier effect contributed by maize decreased disease severity by 28%, and microclimate modification contributed by ryegrass reduced severity by 24% (Fig. 6).

**Fig. 7.**
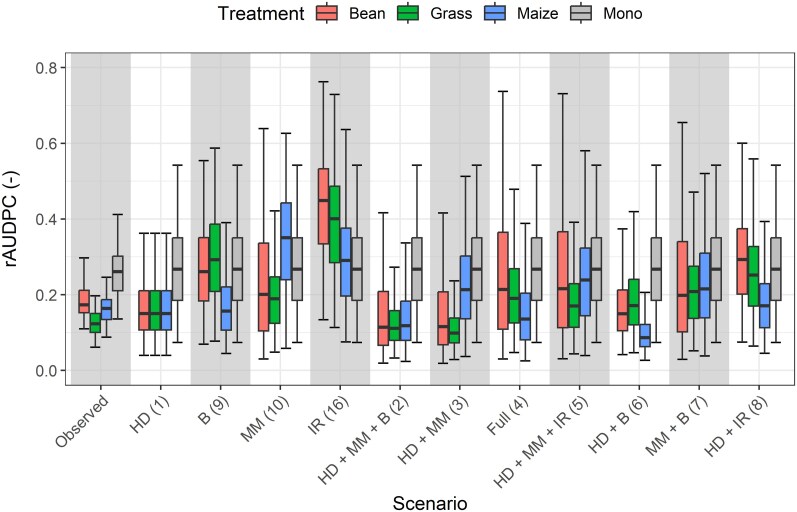
Comparison of observed rAUDPC_obs_ and rAUDPC_sim_ simulated in selected strip-cropping scenarios accounting for uncertainty in the mechanisms of disease suppression. This was accomplished by repeating the simulation with 500 runs per scenario–treatment. rAUDPC_obs_ and rAUDPC_sim_ refer to the relative area under the disease progress curve, for observed and simulated data, respectively. Scenarios were combinations of toggled active mechanisms (HD, B, MM, or IR). The leftmost boxplot depicts observed disease, followed by simulated scenarios with single mechanisms assumed active (HD, B, MM, or IR, respectively). This is followed by the best ranking scenarios until the eighth highest. Refer to Supplementary Fig. S3 for boxplots of the remaining scenarios, including outliers. ‘Baseline’ refers to the scenario with no active mechanisms, thus simulating strip crops identically to monoculture. ‘Full’ refers to the scenario with all four mechanisms active. Numbers in parentheses indicate the ranking that was determined using a log-likelihood analysis of rAUDPC_obs_ compared with rAUDPC_sim_. Mechanism abbreviations: HD (host dilution); MM (microclimate modification); B (barrier effect); IR (induced resistance). Refer to Supplementary Figs S3–S6 for figures of boxplots for each strip-crop treatment under all scenarios.

rAUDPC_sim_ distributions, generated through propagation of uncertainty in the effects of disease-suppressive mechanisms (Fig. 7; Supplementary Figs S3–S6), generally agreed with mean simulated DPCs (Fig. 5). The scenario which combined the effects of host dilution, microclimate modification, and the barrier effect ranked second overall in NLL (instead of first with MAE), following the scenario which only considered the effect of host dilution, which had ranked first with NLL, but fifth with MAE. Nonetheless, comparing distributions visually suggests that the scenario combining host dilution, microclimate modification, and the barrier effect most appropriately detects differences in disease suppression between companion crops, which host dilution alone is unable to predict, given that this mechanism simulates all strip crops identically (since the proportional area and arrangement of host plants does not vary between strip crops). Compared with the ranking by MAE, ranking by NLL slightly worsened the scenarios which included induced resistance. Nonetheless, induced resistance was not included in any of the three best scenarios ranked by MAE or by NLL.

## Discussion

In this study, we quantified the relative importance of four different mechanisms—host dilution, microclimate modification, barrier effect, and induced resistance—potentially driving disease suppression in potato strip-cropped with different companion crops (faba bean, ryegrass, and maize) and compared this with potato grown as a monoculture. By measuring the various mechanisms in the field and subsequently incorporating these in a standard epidemiological model, we were able to quite accurately predict disease progression. Predictions of disease severity in the strip crops were most accurate in the scenario where host dilution, microclimate modification, and barrier effect were simultaneously considered. As hypothesized, the relative importance of each mechanism varied depending on the companion crop introduced.

Host dilution is often proposed as the most important mechanism for disease suppression in intercrops (Finckh *et al*., 2000; Boudreau, 2013; Zhang *et al*., 2019). Reducing the density of the susceptible host, by replacing it with a companion crop, leads to spores landing on non-hosts, rendering them ineffective. In our case study, we estimated that on average 66% of released spores were deposited on hosts in strip crops, compared with the monoculture, which led to an estimated reduction of disease severity by 25% (Fig. 6). In addition to host dilution, which was assumed to be identical for each strip-crop treatment, the other mechanisms can further modify the level of disease suppression from what is achieved through host dilution alone.

Numerous studies show that intercropping can modify the canopy microclimate and suggest that these modifications, especially to relative humidity, play an important role in disease suppression (Boudreau and Mundt, 1992; Boudreau, 1993; Gómez-Rodríguez *et al*., 2003; Boudreau and Shew, 2006; Schoeny *et al*., 2010; Gao *et al*., 2021). In our case study, without consideration of other mechanisms, simulations using only microclimate modifications of the strip crops resulted in a 24% reduction in rAUDPC_sim_ in the strip crop with ryegrass compared with the monoculture, while a 16% increase was simulated for the strip crop with maize. Notably, these large effects on disease progress arise from relatively small differences in microclimate. In July 2022, the daily average relative humidity was 78.9% in the potato monoculture, 77.8% in the potato–grass, 80.6% in the potato–maize, and 79.4% in the potato–faba bean. These small differences result in part from taking a daily average, not taking into account the variation in the daily amplitude of relative humidity across strip crops (Supplementary Fig. S1A). For example, we report insignificant differences in mean hours per day that relative humidity meets thresholds for disease processes in the epidemic model (Table 2); however, when analyzed for shorter time windows, differences were in fact significant (Homulle *et al*., 2025). Even so, it is important to consider that temperature and relative humidity drive many key processes in the disease cycle of *P. infestans*, and other pathogens, including spore germination and infection, lesion growth, and sporulation (Crosier, 1934; Harrison and Lowe, 1989; Zwankhuizen *et al*., 1998). Consequently, seemingly small differences in microclimate may accumulate by influencing various disease processes and over the course of a season, resulting in significant differences in disease severity, which can be either beneficial or detrimental compared with the monoculture.

Similarly, the barrier effect of companions could either promote or suppress disease. We found that the barrier effect was much stronger with maize as a companion than with faba bean or ryegrass. We presume that this is mainly due to its significant height advantage (Supplementary Fig. S7); however, other companion traits, such as leaf area index (LAI), leaf area density (LAD), or leaf angle may also contribute towards the barrier effect (Calonnec *et al*., 2013; Tivoli *et al*., 2013; Vidal *et al*., 2017b; Ahn *et al*., 2020). Potato plants strip-cropped with maize received fewer spores than the monoculture, and as a result simulations considering only the barrier effect estimated a 28% reduction in disease severity in the strip crop with maize compared with the monoculture. In contrast, potato strip-cropped with ryegrass were exposed to a small increase in spore deposition. Faba bean contributed only a slight decrease in spore deposition, despite being taller than potato; however, other canopy characteristics, such as a higher porosity or weaker establishment, may have reduced its effectiveness as a barrier (Homulle *et al*., 2024, 2025). Lastly, the effect of induced resistance simulated higher severities in all strip crops, but more so for the strip crop with faba bean or ryegrass than maize.

Interestingly, no single mechanism could explain the majority of the variation in observed disease levels between the various strip crops. Only when multiple mechanisms were combined could we capture the observed disease severity, suggesting that several mechanisms are indeed important for disease regulation. While host dilution is often recognized as the most important disease-suppressive mechanism, we show that contributions of other mechanisms are of a similar magnitude, and must be considered for evaluating overall suppression. The combinations of mechanisms varied in their capacity to replicate the observed disease progress curves, but, overall, the most successful scenario integrated the effects of host dilution, microclimate modification, and barrier effect. For other pathosystems, the mechanisms we have studied may be of lesser or greater importance, or other mechanisms may be involved.

Disease reduction in intercropping varies widely across studies (Zhang *et al*., 2019; Beillouin *et al*., 2021). Our study suggests that substantial variation in disease suppression can be attributed to the fact that the strength and direction of disease-suppressive mechanisms change based on the choice of the companion crop. The strength of some mechanisms is likely to be driven by traits of the companion, such as height, LAI, and LAD (Vidal *et al*., 2017b; Levionnois *et al*., 2023), and the interplay of these traits and mechanisms can result in varying levels of disease suppression. For example, companion architecture and canopy density both affect spore dispersal and microclimate (Vidal *et al*., 2017a). Notably, disease-suppressive mechanisms are not necessarily complementary, and they can act in opposition. For instance, in the strip crops with ryegrass and maize, the effect of microclimate modification and barrier effect counteracted each other. Maize provided a strong barrier effect, resulting in an estimated 28% reduction in severity. However it increased the duration of high relative humidity in the potato canopy, promoting the establishment and growth of *P. infestans*, and resulting in an estimated 16% increase in severity. Conversely, in the strip crop with ryegrass, potato plants benefitted from reduced relative humidity, resulting in an estimated 24% reduction in severity, yet were exposed to higher spore deposition, leading to a 4% increase in severity. Nevertheless, the net effect of the two mechanisms resulted in disease suppression in both strip-cropping systems.

### Generalizing the approach

Modeling disease-suppressive mechanisms helps us to understand how species mixtures contribute towards disease suppression, demonstrating that different companion crops can reduce disease pressure, even if the mechanisms operate differently. We tested our modeling approach on data from a field trial with a similar setup conducted in 2021 (Supplementary Fig. S8). Simulations of the 2021 epidemic that included canopy microclimate data measured in 2021 and simulated host dilution accurately predicted disease progression in the strip crops. However, when simulations also included the barrier effect estimated from particle deposition measurements of 2022, disease suppression in the strip crop with maize was overpredicted. This overprediction may be due to an overestimation of the barrier effect by using 2022 measurements, because in 2021 maize was planted 4 weeks later than potato, resulting in shorter maize that was probably less effective as a barrier (Supplementary Fig. S7; Homulle *et al*., 2024). Modeling the 2021 season suggests that our approach can be generalized to other years, but the strength of individual mechanisms will depend on characteristics of the companion canopy, that can be sensitive to seasonal effects. By using measured microclimate data, the model captures season-specific variation in weather. However, other mechanisms are probably also sensitive to seasonal variation. For example, early-season disease pressure might over-ride the barrier effect if companion crops have not yet reached an effective height or canopy density. Conversely, in seasons with low disease pressure, disease-suppressive mechanisms may not contribute additional disease control.

This illustrates that the strength of disease-suppressive mechanisms will depend on traits and the epidemiology of the pathogen, and how these interact with the host and companion. Canopy characteristics or companion traits that suppress the dispersal or growth of certain diseases may promote others (Vidal *et al*., 2018; Gao *et al*., 2021). Companion characteristic measurements, such as LAI, height, or leaf angle, could, for example, be incorporated when estimating the strength of the barrier effect. Another consideration is that the phenology of the companion may govern the timing of disease-suppressive effects during the growing season (Schoeny *et al*., 2010; Homulle *et al*., 2025). Additionally, almost all management decisions can affect the disease epidemiology directly or indirectly. A resistant cultivar may affect disease progress through affecting establishment/survival of the pathogen, as well as its proliferation by affecting the canopy structure which in turn may affect microclimate. Testing of our modeling approach under a wider range of conditions or with other relevant pathosystems will improve confidence in our approach, and it may further explain the large observed variation in disease suppression across intercrop systems.

### Application

Our findings point towards the importance of management decisions (such as planting date) for the strength of disease suppression (Levionnois *et al*., 2023). The approach could be used to explore how intercrop design and management affect disease suppression. To calculate the effect of host dilution, our approach uses field dimensions occupied by the host and the companion. Aspects such as strip width could be assessed and potentially optimized (Juventia *et al*., 2022). Other arrangements, such as blocks, could also be explored. For other layouts with closer inter-mixing of canopies, such as row or intra-row mixing, approaches based on 3D plant architecture or principles of light interception could be more applicable (Gigot *et al*., 2014; Vidal *et al*., 2017b; Levionnois *et al*., 2023). However, additional data on microclimate and the barrier effect under various intercropping arrangements, or reliable methods to predict them, would be necessary since the spatial arrangement of the host and companion, in relation to their characteristics, can impact the strength of these mechanisms. For example, as the ratio of maize in pepper–maize and bean–maize intercrops increased, the relative humidity compared with the respective monocultures also increased (Boudreau, 1993; Gao *et al*., 2021). The proportion of maize in an intercrop could also lead to trade-offs with its effectiveness as a barrier and the spatial effect of host dilution on spore dispersal.

Lastly, our approach of modeling disease suppression may also be combined with decision support system models in the context of strip intercropping. This integration could determine a spraying schedule for strip-cropping systems, and assess whether conditions in strip crops allow for less (frequent) spraying or delaying of the first application (Boudreau *et al*., 2016; Yan *et al*., 2024). Ultimately, it could help investigate potential reductions in fungicide application in intercrops, contributing to more sustainable crop protection.

### Model uncertainties

In our approach, estimation of rate modifiers for mechanisms underlying disease suppression of strip intercropping compared with monoculture relies on several simplifying assumptions, especially with respect to spore dispersal. We attempted to isolate the spatial effect of host dilution from the barrier effect, although host dilution implicitly interacts with the particle collection data which were used to estimate the barrier effect, leading to a possible overestimate of disease suppression compared with the monoculture when these mechanisms are combined in simulations. Furthermore, we assumed that the barrier effect was only dependent on wind speed, and wind direction and turbulence were not explicitly considered, but these aspects can impact disease dynamics. For example, in a strip-crop system, the orientation of potato strips with respect to the wind direction affected the observed reduction in late blight, with the greatest reductions in plots planted perpendicular to the wind (Bouws and Finckh, 2008). Wind turbulences may affect the movement of disease propagules, potentially leading to inoculum loss outside the plot, but these patterns are complex and probably change with crop growth stage, architecture of the neighbor, and planting pattern (e.g. strip versus row intercrops; Boudreau and Mundt, 1992; Boudreau, 2013).

The data from the *in vitro* detached leaf assay indicated that defense responses are down-regulated in the intercrops compared with the monoculture, leading to increased infection rates of late blight by 7–64%. These results are inconsistent with typically observed resistance levels (Vleeshouwers *et al*., 1999; Skelsey *et al*., 2009; Kessel *et al*., 2010), suggesting that the assay may have overestimated the impact of companion crops on potato plant resistance. Furthermore, including induced resistance in the model simulations worsened the predictions compared with the observed disease severities, indicating that the magnitude of the effect of induced resistance as observed *in vitro* may not have been present to the same extent in the field. It is noteworthy that when the detached leaf assay was repeated in 2024, no significant differences between treatments was detected (Homulle *et al*., 2025). This highlights the need to further investigate the role of induced resistance in field conditions to better understand its contribution to disease regulation.

While previous studies have reported on disease-suppressive mechanisms in intercrop experiments (Gómez-Rodríguez *et al*., 2003; Schoeny *et al*., 2010; Gao *et al*., 2021), assessing the contribution of individual mechanisms to overall disease suppression can be challenging. Introducing an additional species in intercropping brings a range of mechanisms that may interact synergistically or in opposition. Where other studies attribute changes in disease pressure to differences measured in the crop, such as in relative humidity, we propose that these measurements can be used to quantify the contributions of intercrop companions to disease suppression. By incorporating field measurement in an epidemiological model, we were able to predict disease severity in a strip-crop system. This helped to accomplish disentangling the importance of different mechanisms and quantifying their contribution to disease suppression. Our study has shown, for instance, that small differences in microclimate induced by strip cropping can significantly influence disease severity, effectively quantifying the accumulation over a growing season of the effect of microclimate modifications. This study is the first to our knowledge to use a mechanistic simulation approach to disentangle the mechanisms leading to disease suppression in strip-cropping systems.

## Conclusion

This study describes a novel approach used to model the effects of strip-crop-related disease-suppressive mechanism, using late blight in potato as a case study. Field data were used to estimate parameters for host dilution, microclimate modification, induced resistance, and barrier effects. These were then integrated into a mechanistic late blight simulation model, which characterizes the effect of environmental variables on host–pathogen interactions. The model predicts that even small differences in microclimate as induced by different companion crops can significantly affect disease severity. The severity of disease in strip-cropping systems could only be reproduced by a combination of mechanisms, suggesting that all those mechanisms are relatively important. This methodology sets a premise for modeling the effects of strip cropping on disease dynamics, as well as the development of other approaches that may be used to further study and optimize disease suppression in intercrop systems.

## Supplementary Material

erag097_Supplementary_Data

## Data Availability

The primary data supporting this study were not made publicly available at the time of publication. The data that support the findings of this study are available from the corresponding author upon request.

## Supplementary data

The following supplementary data are available at *JXB* online.

Protocol S1. Modifications to the BLIGHTTIME model.

Protocol S2. Simulation of the effect of host dilution.

Fig. S1. Mean hourly canopy relative humidity and temperature for monoculture and strip-crop treatments in July 2022.

Table S1. Generalized linear mixed effect model coefficients used to estimate effect multipliers for the effect of companion crops on deposition efficiency and infection efficiency.

Fig. S2. Disease progress curves (DPCs) for all scenarios.

Fig. S3. Comparison of observed rAUDPC_obs_ and rAUDPC_sim_ simulated in all strip-cropping scenarios accounting for uncertainty in the mechanisms of disease suppression.

Fig. S4. Comparison of observed rAUDPC_obs_ and rAUDPC_sim_ simulated for potato strip cropped with faba bean across all strip-cropping scenarios accounting for uncertainty in the mechanisms of disease suppression.

Fig. S5. Comparison of observed rAUDPC_obs_ and rAUDPC_sim_ simulated for potato strip cropped with ryegrass across all strip-cropping scenarios accounting for uncertainty in the mechanisms of disease suppression.

Fig. S6. Comparison of observed rAUDPC_obs_ and rAUDPC_sim_ simulated for potato strip cropped with maize across all strip-cropping scenarios accounting for uncertainty in the mechanisms of disease suppression.

Fig. S7. Height of potato and companion crops in 2021 and 2022.

Fig. S8. Disease progress curves (DPCs) simulated for strip-crop scenarios in 2021.

## Abbreviations

AUDPC: area under the disease progress curve
MAE: mean absolute error
NLL: negative log-likelihood
