# Supplementary material for: Quantifying the relative importance of disease-suppressive mechanisms in species mixtures: a case study of late blight in strip-intercropped potato
Source: J Exp Bot. 2026 Feb 24;77(12):3939–54. doi: 10.1093/jxb/erag097 (PMC13293120; doi:10.1093/jxb/erag097)
Supplement: erag097_Supplementary_Data [file erag097_supplementary_data.pdf]

Journal of Experimental Botany **Supplementary data**

Article title: Quantifying the relative importance of disease suppressive mechanisms in species mixtures: a case study of late blight in strip-intercropped potato

Authors: Matthew Brandon, Zohralyn Homulle, Jacob C. Douma

Article acceptance date: 09 February 2026

The following Supplementary data is available for this article:

Protocol S1 Modifications to the BLIGHTTIME model

Protocol S2 Simulation of the effect of host dilution

Fig. S1 Mean hourly canopy relative humidity (a) and temperature (b) for monoculture ('mono') and strip-crop treatments in July 2022

Table S1 Generalized linear mixed effect model coefficients used to estimate effect multipliers for the effect of companion crops on deposition efficiency and infection efficiency

Fig. S2 Disease progress curves (DPCs) for all scenarios

Fig. S3 Comparison of observed rAUDPCobs and rAUDPCsim simulated in all strip-cropping scenarios accounting for uncertainty in the mechanisms of disease suppression

Fig. S4 Comparison of observed rAUDPCobs and rAUDPCsim simulated for potato strip cropped with faba bean across all strip-cropping scenarios accounting for uncertainty in the mechanisms of disease suppression

Fig. S5 Comparison of observed rAUDPCobs and rAUDPCsim simulated for potato strip cropped with ryegrass across all strip-cropping scenarios accounting for uncertainty in the mechanisms of disease suppression

Fig. S6 Comparison of observed rAUDPCobs and rAUDPCsim simulated for potato strip

cropped with maize across all strip-cropping scenarios accounting for uncertainty in the mechanisms of disease suppression

Fig. S7 Height of potato and companion crops in 2021 and 2022

Fig. S8 Disease progress curves (DPCs) simulated for strip-crop scenarios in 2021

Protocol S1 Modifications to the BLIGHTTIME model

The 'BLIGHTTIME' model (Skelsey et al., 2009) was used to simulate microclimate-dependent late blight epidemics. Slight modifications were made to the original model, including: a leaf wetness infection requirement (blight hour function), a maximum lesion number per plant, a maximum lesion age, temperature-dependent latency progression and high-temperature lesion inactivation. Additionally, the structure of the model was modified to simultaneously track the number of lesions produced at each times step, and the area that is divided across various stages of infection (latency, infectious, non-infectious) of lesions produced at each time step.

The original BLIGHTTIME model uses a Leslie matrix approach, keeping track of the number and radius of lesions of all ages for each time step. Lesions are transitioned through consecutive age classes, which account for the duration of latency and infectiousness of lesions. We modified the structure of the model to a compartmental approach, drawing inspiration from BLIGHTSIM (Narouei-Khandan et al., 2020a, 2020b). Instead of complete transition of lesion (age) classes to consecutive classes at each time step, lesion area progresses through classes at a variable rate. This allows for a variable, microclimate-dependent latency period. Lesions classes LAT1-LAT5, INF, and NINF represent how the area of each lesion is divided across all lesion ages classes. LAT1-LAT5 approximately corresponds to individual days of a five-day latency (although the microclimate-dependent latency progression rate can accelerate or prolong the period that lesion area remains in each category). INF stores the lesion area that is infectious, while NINF stores the lesion area that is no longer infectious. A separate vector records the number of lesions initiated at each time step, which is used for functions which act on whole individual lesions.

Lesion growth rate (LGR) determines the rate of radial expansion of lesions; calculated lesion growth is removed from healthy leaf tissue (H) and added to the first latent class (LAT1). Individual lesions can only grow to the border of a leaflet, which is modeled as a circle with a set radius of 0.03 m; development of a lesion continues until its corresponding area has fully progressed to NINF. Latency progression rate (LPR) defines the rate of transition of area from LAT1 through the consecutive LAT compartments and into INF. Under optimal LPR conditions 1/24th of each latent compartment is progressed to the next compartment at each hourly time step. Lesion area sporulates for as long as it remains in the INF compartment, before progressing to the NINF compartment. A modified function for lesion death

(described below) can accelerate the removal from LAT and INF compartments, by inactivating whole lesions and thus any corresponding lesion area (across all stages LAT, INF, NINF) in these compartments; this process is separate from hourly INF inactivation, which progresses 1/24th of INF area to NINF at each time step, accounting for the approximate one-day infectious period of lesion area.

Spore production is calculated as a function of INF area. All spores are pooled and immediately dispersed and deposited for each time step during which sporulation occurs (according to microclimate conditions and the presence of infectious lesion area). The rate of spore deposition at each time step is determined by the deposition efficiency (DE). Deposition is a prerequisite for the initiation of a new lesion. Lesions can only be initiated if the blight hour requirement is met (the blight hour function is described below), and if so, the infection rate of spores resulting in a new lesion is determined by the infection efficiency (IE). At the end of each time step, spores that failed to initiate a lesion are permanently removed.

Blight hour function

This function relates to the temperature-dependent requirement of extended leaf wetness for infection to occur (Crosier, 1934). It is a modification of the blight day function of the original BLIGHTTIME model, which specifies that a minimum number of consecutive "infection-hours" with RH > 90% and temperature between 10 and 27°C must be met for infection to be able to occur on a given day. The minimum number of hours that is required for a blight day depends on the average temperature over the consecutive infection-hours (Rotem et al., 1970; Zwankhuizen & Zadoks, 2002). An approach similar to that of the Nærstad model was used, which calculates infection risk at any given hour contingent on microclimate conditions in the following hours (Hjelkrem et al., 2021).

The following operation is performed for every hour to determine whether it is a 'blight hour' in which infection may occur. Including the starting hour, at least three consecutive leaf wetness are required for infection, however, as average temperature deviates from the optimal range of 15 to 20°C, the required duration increases (Hartill et al., 1990; Rotem et al., 1970). Leaf wetness is assumed for any hour with RH ≥ 87%. If the first three-hour consecutive hours of leaf wetness do not meet the temperature requirement, additional hours of leaf wetness are no longer consecutively required, but within an 11-hour interval, a sufficient

threshold of hours of leaf wetness must be met, determined by the average temperature over the entire period. To qualify the hour at the beginning of the period as a blight hour, the average temperature over the duration of leaf wetness hours must fall above the line shown in Fig. 1

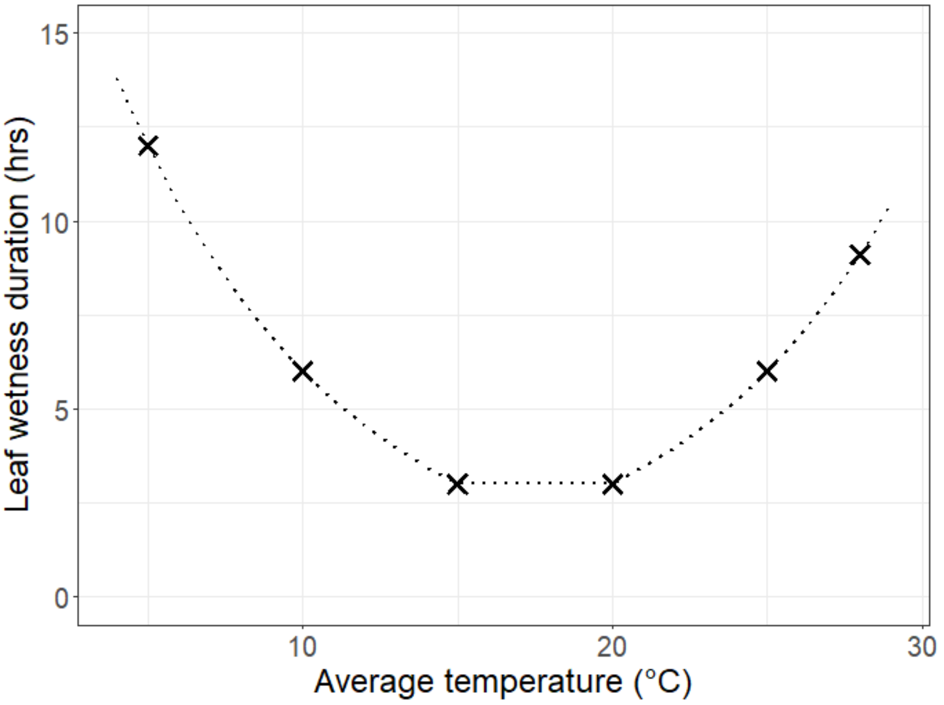

Fig. 1 Duration of leaf wetness and average temperature required for *P. infestans* germination as modified for the late blight epidemic model (Rotem et al., 1970). As optimal temperatures for germination (15-18°C) are approached, leaf wetness duration requirements become less stringent and vice versa.

High-temperature lesion inactivation

Pre-existing late blight models rarely include functions which inactivate lesions under high temperature and/or low humidity conditions. Quantified relationships between these conditions and lesion survival are not well described, although qualitative observations have been made (Crosier, 1934). LATEBLIGHT inactivates all lesions if daily mean temperature exceeds 41°C for three consecutive days (Wallin & Hoyman, 1958). An alternative method is proposed for this study, although it was not experimentally tested. Lesion death rate, LD_t, is calculated as the proportion of lesions which inactivate at each time step depending on the time step temperature:

$$LD_t = 1 - \left[\frac{1}{1 + \exp(T_t - T_{thres})}\right]^{\Delta t}$$

with T_t as the temperature (°C) at time t and Δt as the duration of a time step (1 hour). T_{thres} is the temperature at which $LD = 0.5$ if T_{thres} is maintained for 24 hours.

All pre-existing functions are factored by 1/24 to convert daily rates to hourly rates, but such an approach for LD would imply that a maximum limit of 1/24 lesions can be killed at any hour. To overcome this limitation, the proposed relationship raises survival rate to the power of Δt and takes the difference from 1 as the death rate. Thus, at 30°C, $LD \approx 0$, while at 37°C, $LD \approx 0.15$ for one hour (Fig. 2).

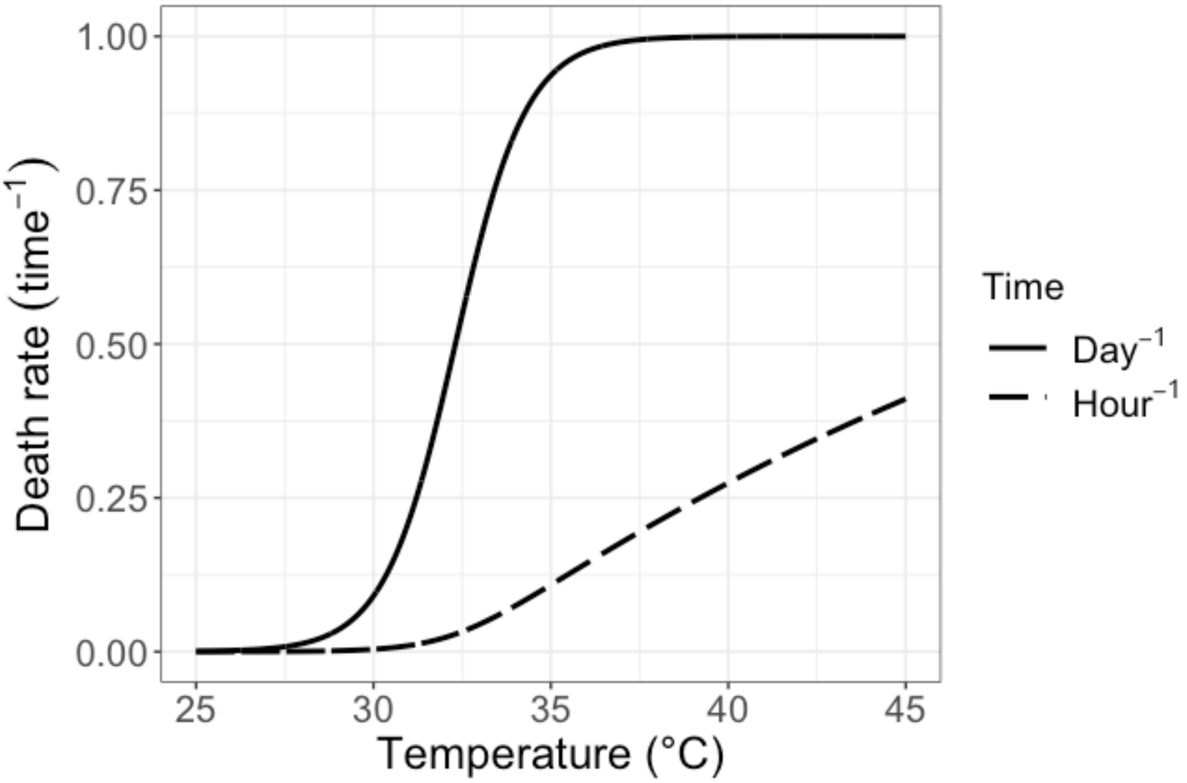

Fig. 2 The total number of active lesions is reduced by LD and the area of these lesions allocated to LAT and INF compartments is moved to the NINF compartment. Additionally, if temperature exceeds 34°C during the 24 hours following initiation of new lesions, they will inactivate (Crosier, 1934; Wallin & Hoyman, 1958). This condition is checked at the time step of lesion initiation, and these inactivated lesions will not contribute towards the total lesion number. Any inactivated lesion will no longer continue to grow or develop.

Latency progression rate

The modification of the original BLIGHTTIME structure from a Leslie matrix to a compartmental structure was done to allow for variable latency progression. The age classes of the BLIGHTTIME model were originally structured by hour but were converted to day compartments L1-L5. The latency progression rate modifies the rate that area moves between compartments relative to an unmodified rate $LPR = 1$, which should theoretically transfer each lesion age category to the next stage. That being said, the modified version transfers 1/24th of each compartment at each time step; thus transfer is in fact modeled as an exponential decay with a decay factor of 1/24, which is modified by the temperature-dependent LPR.

Maximum lesion age

As a result of the addition of a variable latency progression rate, and the accompanied compartmental lesion area tracking, an individual lesion can never progress through all stages (latent, infectious, non-infectious) completely (although area will approach zero). To account for this, and the slightly retarded progression due to the exponential decay, individual lesions are inactivated 15 days after initiation, as was done in LATEBLIGHT (Arneson et al., 1993; Fry & Bruhn, 1980).

Maximum lesion number

A maximum lesion number per plant is set, using a logistic relationship relating deposition to lesion number; as lesion number increases DE is reduced according to the relationship:

$$DE_{red,t} = \frac{n_{les,max} - n_{les,t}}{n_{les,max}}$$

with DE_{red} as the reduction factor (0: no deposition can occur; 1: DE can take maximum value), n_t is the number of lesions at time t, and $n_{les,max}$ is the maximum lesion number.

Protocol S2 Simulation of the effect of host dilution

The effect of host dilution on spore dispersal was simulated with a static, spatially explicit simulation model. Individual host plants are represented in a grid as they are arranged in the field, in either monoculture or strip-crop arrangements. Subsequently, each host plant releases spores according to identical dispersal kernels superimposed across the entire grid, and the distribution of deposited spores across the grid are evaluated to estimate the percentage of spores that land on either 1) host plants, 2) non-host (companion) plants, or 3) escape the plot.

Two dispersal kernels were analyzed in the estimation of the effect of host dilution on spore dispersal: a radial Laplace and a radial Gaussian kernel. Cross-sections of the dispersal kernels (centered on each host plant) are shown in Fig. 1A. Ultimately, it was chosen to use the radial Laplace kernel, as this kernel was similarly applied in the previous analysis of Skelsey et al. (2005). Furthermore, comparison between the two dispersal kernels in their estimation of the effect of host dilution shows relatively small differences (Fig. 1B).

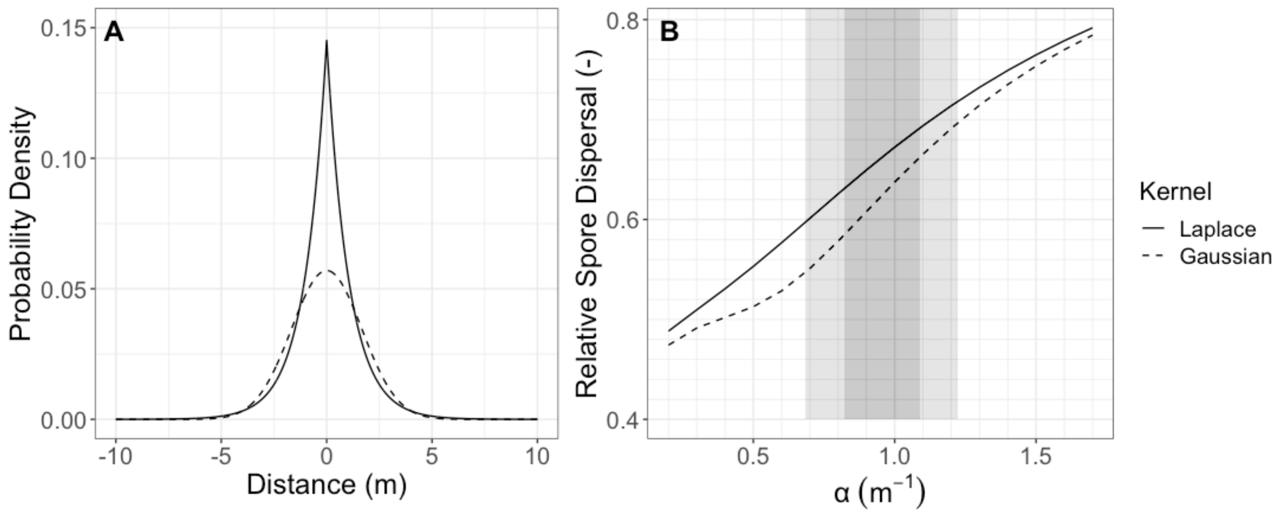

Fig. 1 Dispersal kernels tested for the estimation of the host dilution effect of intercrops. (A) Cross-section of 2-dimensional Gaussian and Laplacian dispersal kernels with α=0.955 m^{-1}. (B) Relative spore deposition simulated in the strip-crop grid compared to monoculture grid across a range of α from 0.2 to 1.7 m^{-1}. The relative spore deposition for a Laplacian kernel was ultimately used to estimate the effect of host dilution. The distribution of effect multipliers for host dilution was simulated from a uniform distribution of α ranging from 0.685 to 1.225 m^{-1}, shaded in light-gray. This sampled range is double the range of α reported

in literature, 0.82 to 1.09 m^{-1}, shaded in dark-gray (Fry & Paysour, 1983).

See Fig. 2 for a graphical representation of the spore dispersal across monoculture and strip-crop plots. In the monoculture grid, using a dispersal gradient of $\alpha = 0.955\ m^{-1}$, 88% of released spores landed on potato cells, while the remaining 12% escaped the bounds of the grid. For the strip-crop grid, 58.3% of released spores landed on potato plant grid cells, 37.2% of spores landed on non-host cells, and the remaining 7.2% escaped the plot. Thus, for the strip-crop and monoculture plots, respectively, 58.3% and 88% represent the proportion of released spores that land on hosts (versus landing on non-hosts or escaping the plot). These values are used as the estimate of the rate of spore deposition in strip-crop and monoculture plots. The effect of host dilution was calculated as the ratio between these deposition rates, re-scaling the relative spore deposition in the strip-crop to 66% compared to the monoculture. Notably, this is only for $\alpha = 0.955\ m^{-1}$ under the given experimental plot dimensions. This calculation was repeated across a gradient $\alpha \sim U(0.685, 1.225)$ [m^{-1}] resulting in relative spore deposition ranging from approximately 0.60 to 0.72 in the strip crops relative to the monoculture. This distribution was sampled for multipliers for the effect of host dilution on spore dispersal and deposition.

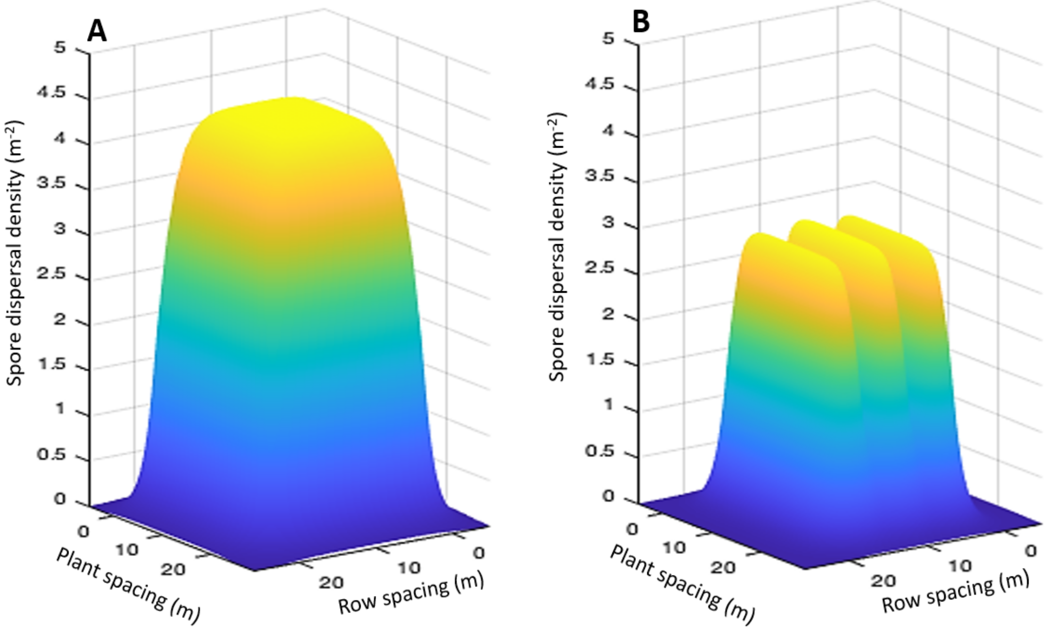

Fig. 2 Surface plots of spatial spore dispersal density in A) monoculture, and B) strip-crop plots. The Laplacian dispersal kernel was used to generate these density surfaces, with α=0.955 m^{-1}. Individual plants were homogenously modelled as spore sources in sole and strip crop arrangements, matching the spatial arrangement of the experimental plots. Integration was used to calculate the cumulative spore deposition across the plot grid and for

host and non-host strips in the strip-cropping system. The figures were generated using MATLAB version R2023a.

Figure S1 Mean hourly canopy relative humidity (a) and temperature (b) for monoculture ('mono') and strip-crop treatments in July 2022

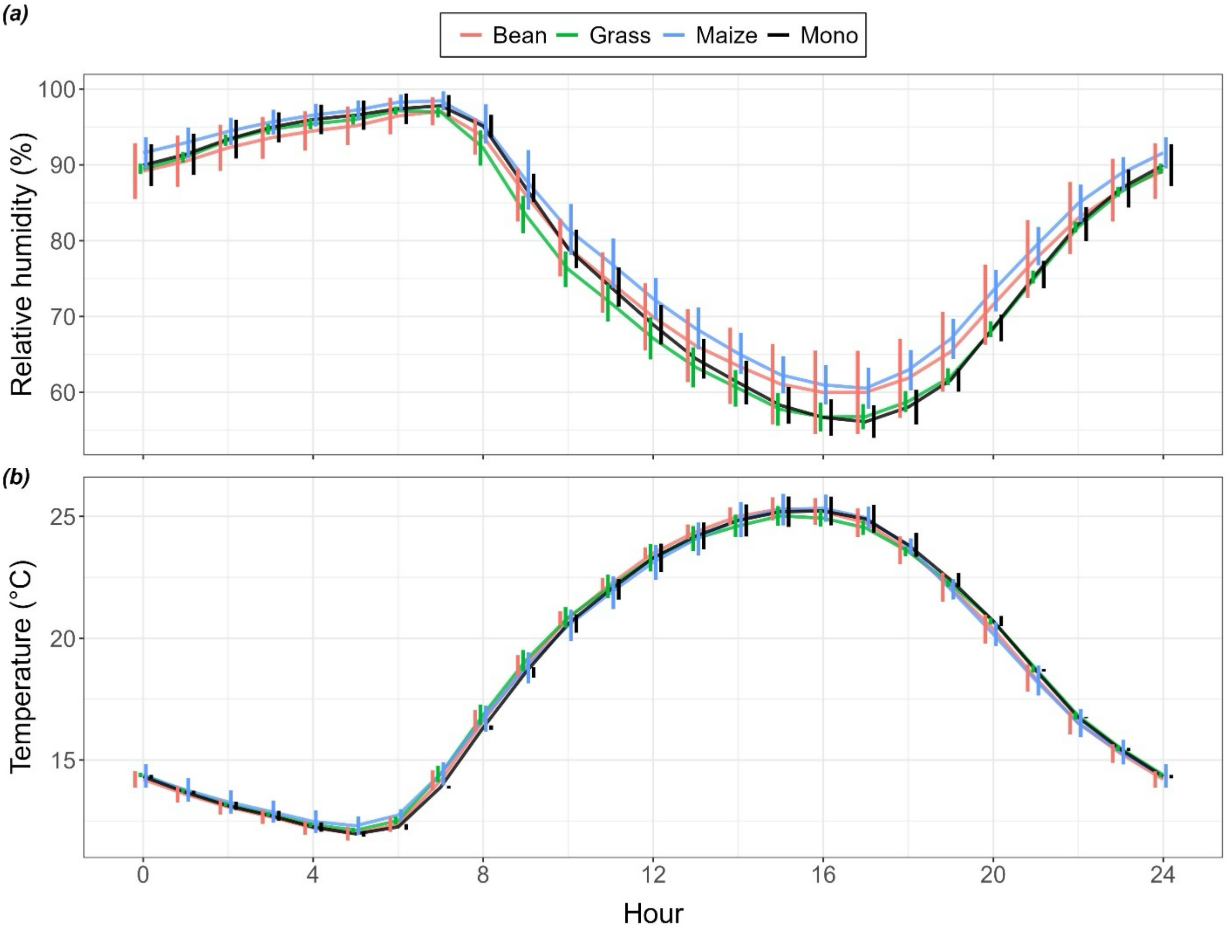

Fig. S1 Mean hourly canopy relative humidity (a) and temperature (b) for monoculture ('mono') and strip-crop treatments in July 2022. Relative humidity (RH) and temperature were measured at 10-minute intervals for n=6 microclimate time series replicates in potato-faba bean ('bean'), potato-ryegrass ('grass'), and potato-maize ('maize') plots (2 replicates per plot, for three plots each) and for n=3 microclimate time series for mono (1 replicate per plot, for three plots). Hourly means represent the mean temperature for a given hour across every day in July 2022. The month of July is used to highlight differences in relative humidity (RH) and temperature between treatments as this was the period when most experimental observations of late blight were made. Curves connecting hourly means are interpolations to improve readability of the figures. Error bars denote ± 1 SD of the mean hourly RH or temperature between replicates.

Table S1. Generalized linear mixed effect model coefficients used to estimate effect multipliers for the effect of companion crops on deposition efficiency and infection efficiency. Only the coefficients necessary for calculating the effect multipliers (according to Eqns. 1 and 2) are shown, with subscript i indicating the companion crop treatment. Coefficients for estimating the barrier effect are on the log scale and both are companion-specific. Coefficients for estimating induced resistance are on the logit scale; the intercept coefficient is the logit-transformed infection rate observed in the monoculture treatment, which is necessary to calculate the relative infection rate in the strip crops in combination with the companion-specific treatment coefficient. †, P < 0.1; *, P < 0.05; **, P < 0.01; ***, P < 0.001

**Mechanism**	**Parameter**			**Bean**	**Grass**	**Maize**	**Mono**
Barrier effect	GLMM coeff.	Intercept: treatment	$\boldsymbol{\beta_0}$	2.715***			
		Slope: treatment	$\boldsymbol{\beta_{1i}}$	0.063	0.127†	-0.238**	0
			$\mathbf{SE}_{\boldsymbol{\beta_{1i}}}$	0.072	0.072	0.075	0
		Intercept: wind speed	$\boldsymbol{\beta_2}$	0.017			
		Slope: treatment-wind speed interaction	$\boldsymbol{\beta_{3i}}$	-0.177**	-0.101	-0.377***	0
			$\mathbf{SE}_{\boldsymbol{\beta_{3i}}}$	0.067	0.067	0.077	0
	Effect multiplier (mean ± SD)			0.96 ± 0.08	1.07 ± 0.09	0.64 ± 0.06	1.00
Induced resistance	GLMM coeff.	Intercept: treatment	$\boldsymbol{\beta_0}$	-0.7885**			
			$\mathbf{SE}_{\boldsymbol{\beta_0}}$	0.248			
		Treatment	$\boldsymbol{\beta_{1i}}$	0.833***	0.596**	0.112	0
			$\mathbf{SE}_{\boldsymbol{\beta_{1i}}}$	0.226	0.229	0.234	0
	Effect multiplier (mean ± SD)			1.64 ± 0.18	1.45 ± 0.18	1.07 ± 0.17	1.00

Figure S2 Disease progress curves (DPCs) for all scenarios

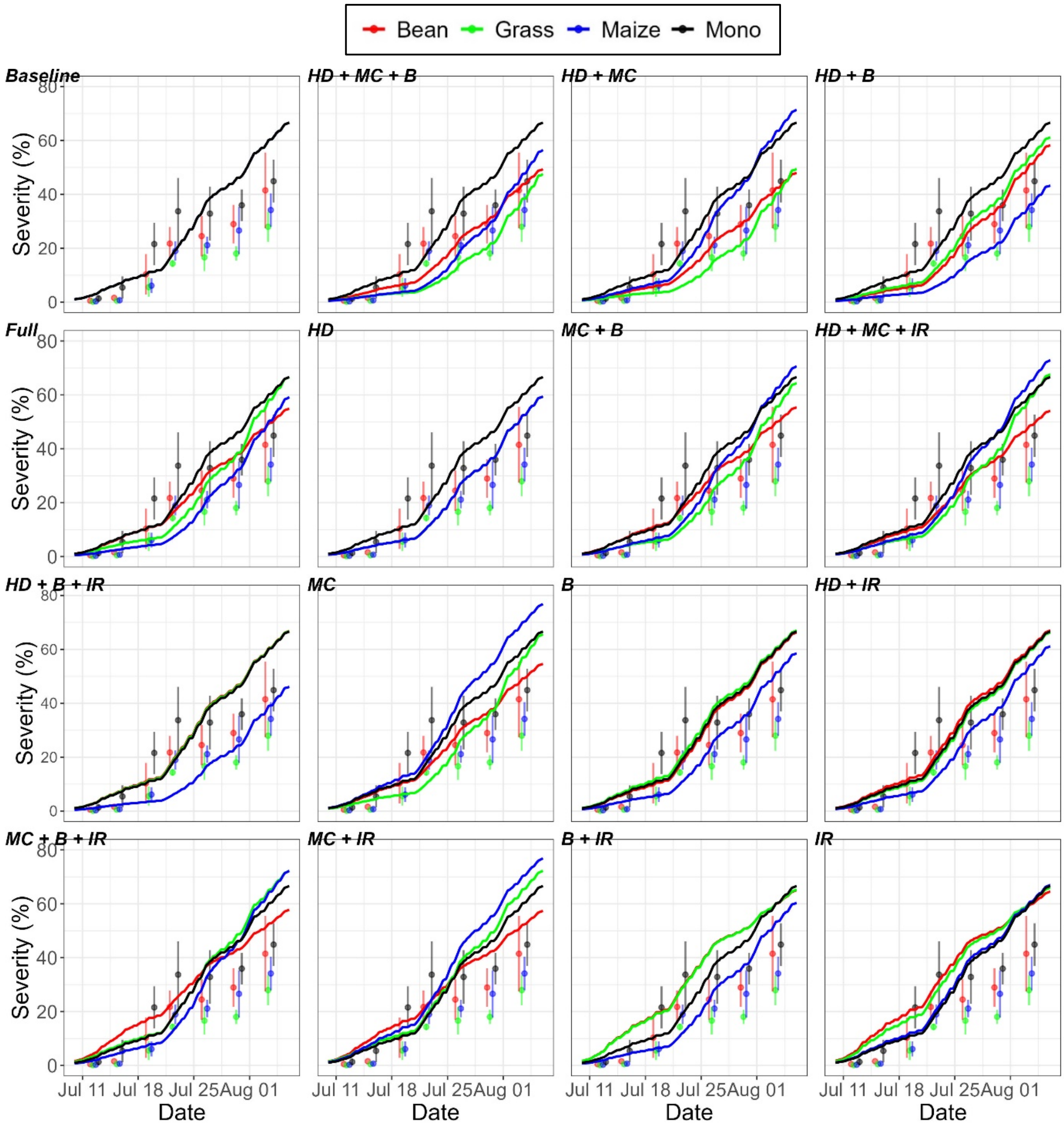

Fig. S2 Comparison of simulated and observed severity across all strip-crop scenarios. For each plot panel, lines represent mean simulated disease progress curves (DPCs) for each treatment under the given scenario. Scattered points with error bars represent the observed mean severity ± 1 SD, which was assessed seven times between 11 July and 4 August 2022 for randomly selected plants from four replicate plots of each treatment. Labels in the upper-left corner of plot panels indicate the scenario. Mechanism abbreviations: HD (host dilution); MM (microclimate modification); B (barrier effect). The ‘baseline’ scenario corresponds to the simulation of disease

progress in the monoculture, and the strip-crop scenario with all strip-crop mechanisms inactivated. Following the 'baseline' plot panel, panels are ordered from left-to-right, and top-to-bottom in order of increasing mean absolute error (MAE) calculated from mean simulated DPCs and observed disease severity (i.e., 'HD + MC + B' is the highest-ranking scenario in MAE, and 'IR' is the lowest-ranking scenario). 'Baseline' was included out of order, as the monoculture reference; this scenario ranked 12th in MAE, after scenario 'HD + IR'.

Figure S3 Comparison of observed rAUDPCobs and rAUDPCsim simulated in all strip-cropping scenarios accounting for uncertainty in the mechanisms of disease suppression.

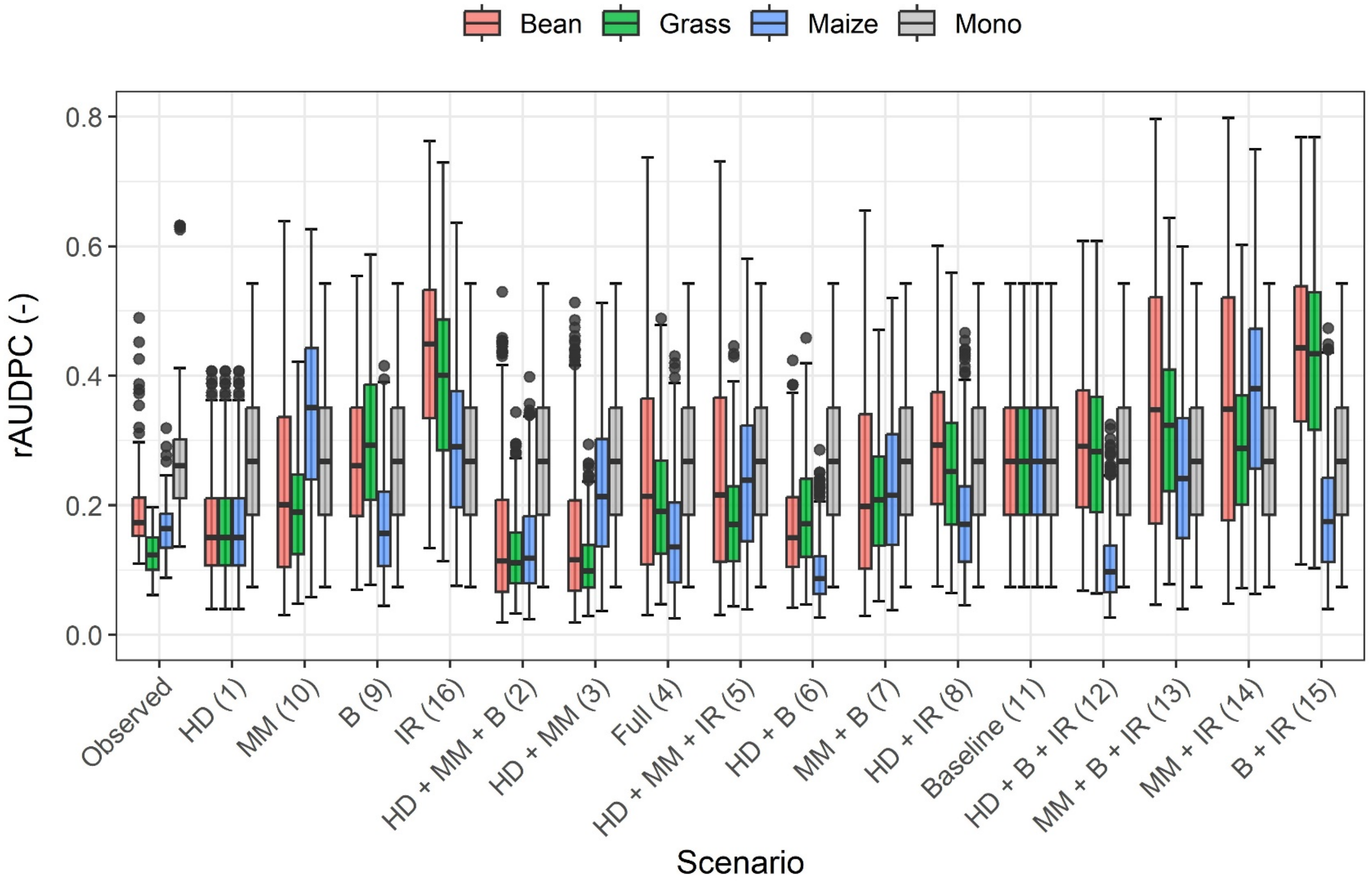

Fig. S3 Comparison of observed rAUDPCobs and rAUDPCsim simulated in all strip-cropping scenarios accounting for uncertainty in the mechanisms of disease suppression. This was accomplished by repeating the simulation with 500 runs per scenario-treatment.

$rAUDPC_{obs}$ and $rAUDPC_{sim}$ refer to the relative area under the disease progress curve, for observed and simulated data, respectively. Scenarios were combinations of toggled active mechanisms (HD, B, MM or IR). The leftmost boxplot depicts observed disease, followed by simulated scenarios with single mechanisms assumed active (HD, B, MM or IR respectively). This is followed by the remaining scenarios, ranked (indicated in brackets) using a log-likelihood analysis of $rAUDPC_{obs}$ compared to $rAUDPC_{sim}$. 'Baseline' refers to the scenario with no active mechanisms, thus simulating strip crops identically to monoculture. 'Full' refers to the scenario with all four mechanisms active. Mechanism abbreviations: HD (host dilution); MM (microclimate modification); B (barrier effect); IR (induced resistance).

Figure S4 Comparison of observed $rAUDPC_{obs}$ and $rAUDPC_{sim}$ simulated for potato strip cropped with faba bean across all strip-cropping scenarios accounting for uncertainty in the mechanisms of disease suppression

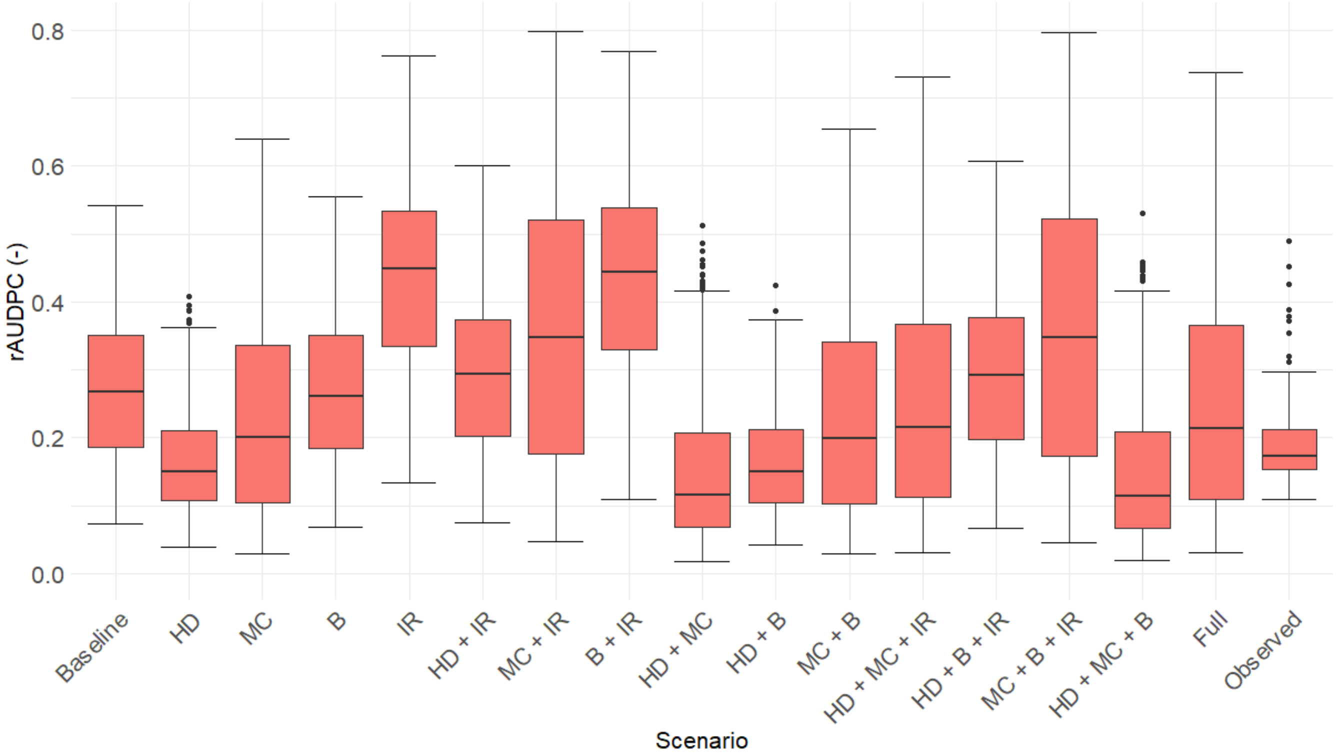

Fig. S4 Comparison of observed $rAUDPC_{obs}$ and $rAUDPC_{sim}$ simulated for potato strip cropped with faba bean across all strip-cropping scenarios accounting for uncertainty in the mechanisms of disease suppression (500 runs per scenario). Scenarios were combinations of toggled active mechanisms (HD, B, MM and/or IR respectively). The 'Observed' boxplot represents rAUDPC which were calculated from observed disease severity, which was assessed between 11 July and 4 August 2022 for randomly selected plants from four replicate plots of each treatment. 'Baseline' represents the scenario in which no disease-suppressive mechanisms are active, and disease in the strip-crops is identical to the monoculture. 'Full' represents the scenario in which all mechanisms are active. Mechanism abbreviations: HD (host dilution); MM (microclimate modification); B (barrier effect); IR (induced resistance).

Figure S5 Comparison of observed $rAUDPC_{obs}$ and $rAUDPC_{sim}$ simulated for potato strip cropped with ryegrass across all strip-cropping scenarios accounting for uncertainty in the mechanisms of disease suppression

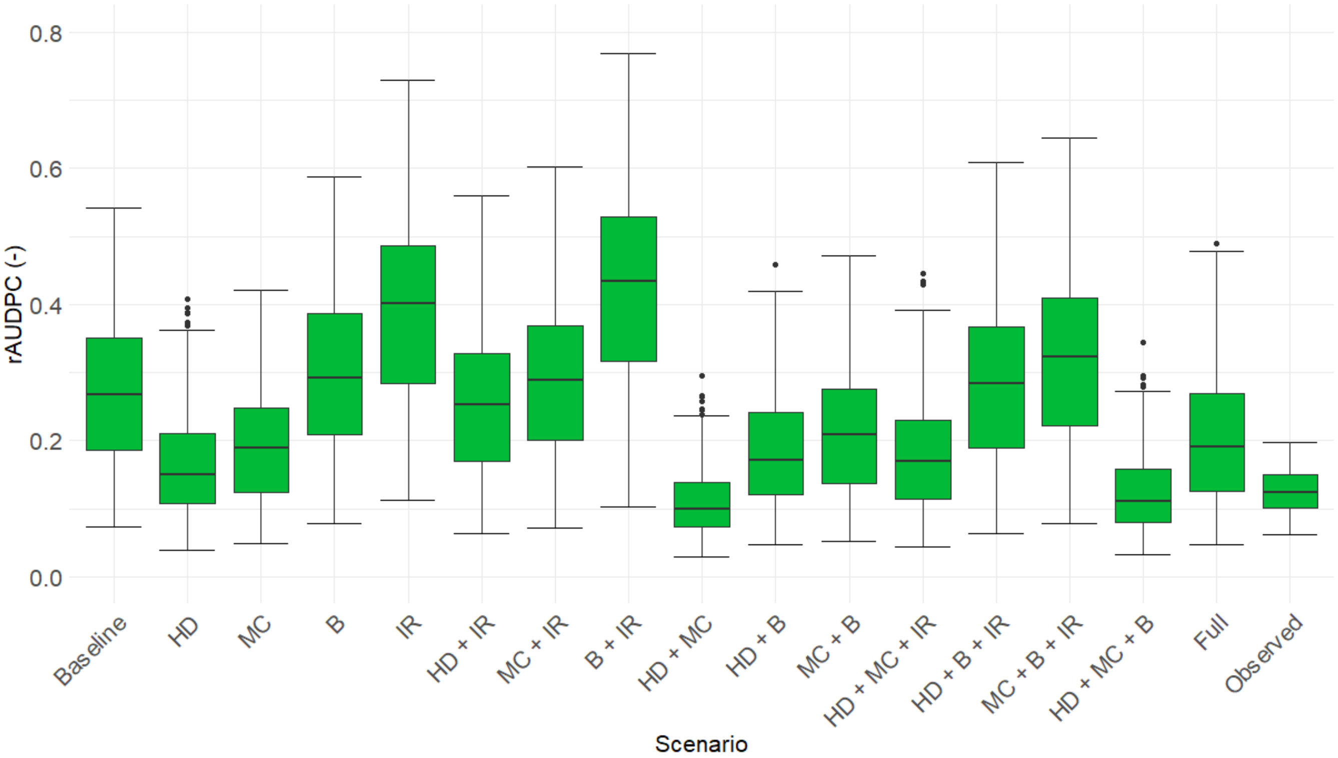

Fig. S5 Comparison of observed $rAUDPC_{obs}$ and $rAUDPC_{sim}$ simulated for potato strip cropped with ryegrass across all strip-cropping scenarios accounting for uncertainty in the mechanisms of disease suppression (500 runs per scenario). Scenarios were combinations of toggled active mechanisms (HD, B, MM and/or IR respectively). The 'Observed' boxplot represents rAUDPC which were calculated from observed disease severity, which was assessed between 11 July and 4 August 2022 for randomly selected plants from four replicate plots of each treatment. 'Baseline' represents the scenario in which no disease-suppressive mechanisms are active, and disease in the strip-crops is identical to the monoculture. 'Full' represents the scenario in which all mechanisms are active. Mechanism abbreviations: HD (host dilution); MM (microclimate modification); B (barrier effect); IR (induced resistance).

Figure S6 Comparison of observed $rAUDPC_{obs}$ and $rAUDPC_{sim}$ simulated for potato strip cropped with maize across all strip-cropping scenarios accounting for uncertainty in the mechanisms of disease suppression

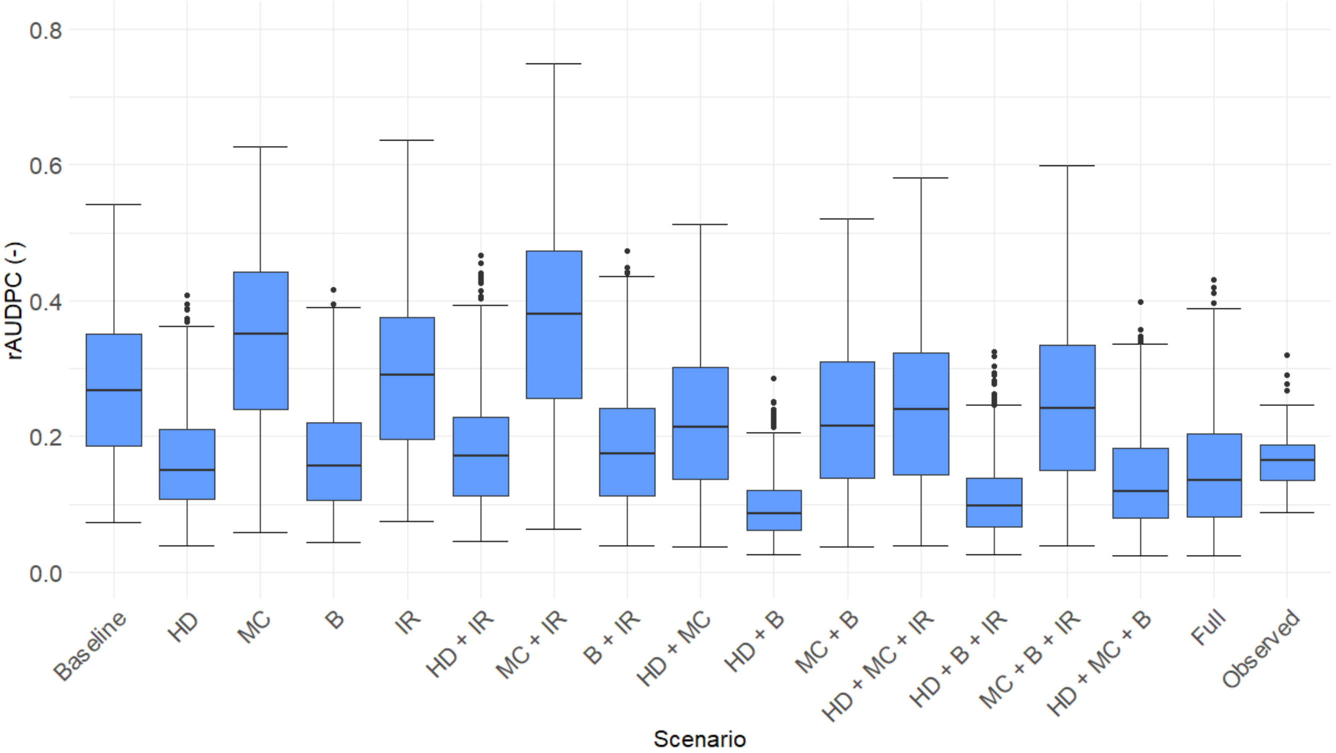

Fig. S6 Comparison of observed $rAUDPC_{obs}$ and $rAUDPC_{sim}$ simulated for potato strip cropped with maize across all strip-cropping scenarios accounting for uncertainty in the mechanisms of disease suppression (500 runs per scenario). Scenarios were combinations of toggled active mechanisms (HD, B, MM and/or IR respectively). The ‘Observed’ boxplot represents rAUDPC which were calculated from observed disease severity, which was assessed between 11 July and 4 August 2022 for randomly selected plants from four replicate plots of each treatment. ‘Baseline’ represents the scenario in which no disease-suppressive mechanisms are active, and disease in the strip-crops is identical to the monoculture. ‘Full’ represents the scenario in which all mechanisms are active. Mechanism abbreviations: HD (host dilution); MM (microclimate modification); B (barrier effect); IR (induced resistance).

Figure S7 Height of potato and companion crops in 2021 and 2022

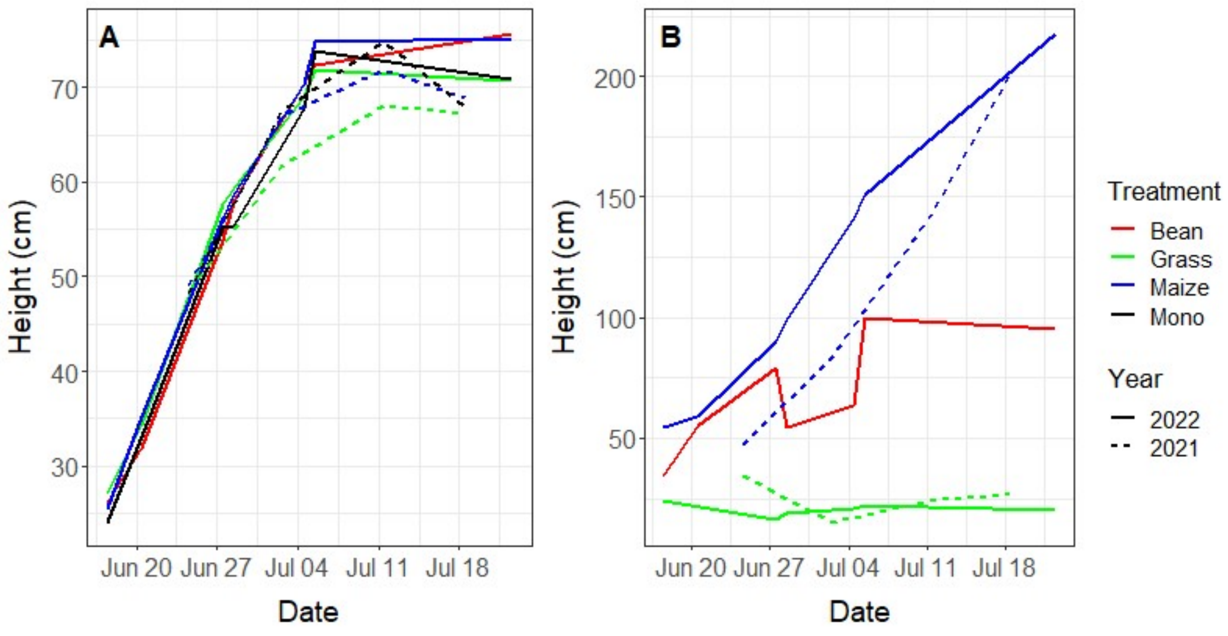

Fig. S7 Mean height of potato and companion crops in 2021 and 2022. In 2021, there were two plots of each treatment, monoculture potato, and potato strip-cropped with ryegrass or maize. In 2022, there were four plots of each treatment: monoculture potato, and potato strip-cropped with faba bean, ryegrass, or maize. The height of potato plants and companion plants were measured across all plots from June 24 to July 18 in 2021, and from June 17 to July 22 in 2022. (A) Height of potato plants planted in monoculture or strip-cropped with different companions. (B) Height of the companion plants of potato in strip-cropped plots. Note that potato was only strip-cropped with faba bean in 2022, not in 2021. In both 2021 and 2022 late blight symptoms were first observed in the field on July 8.

Figure S8 Disease progress curves (DPCs) simulated for strip-crop scenarios in 2021

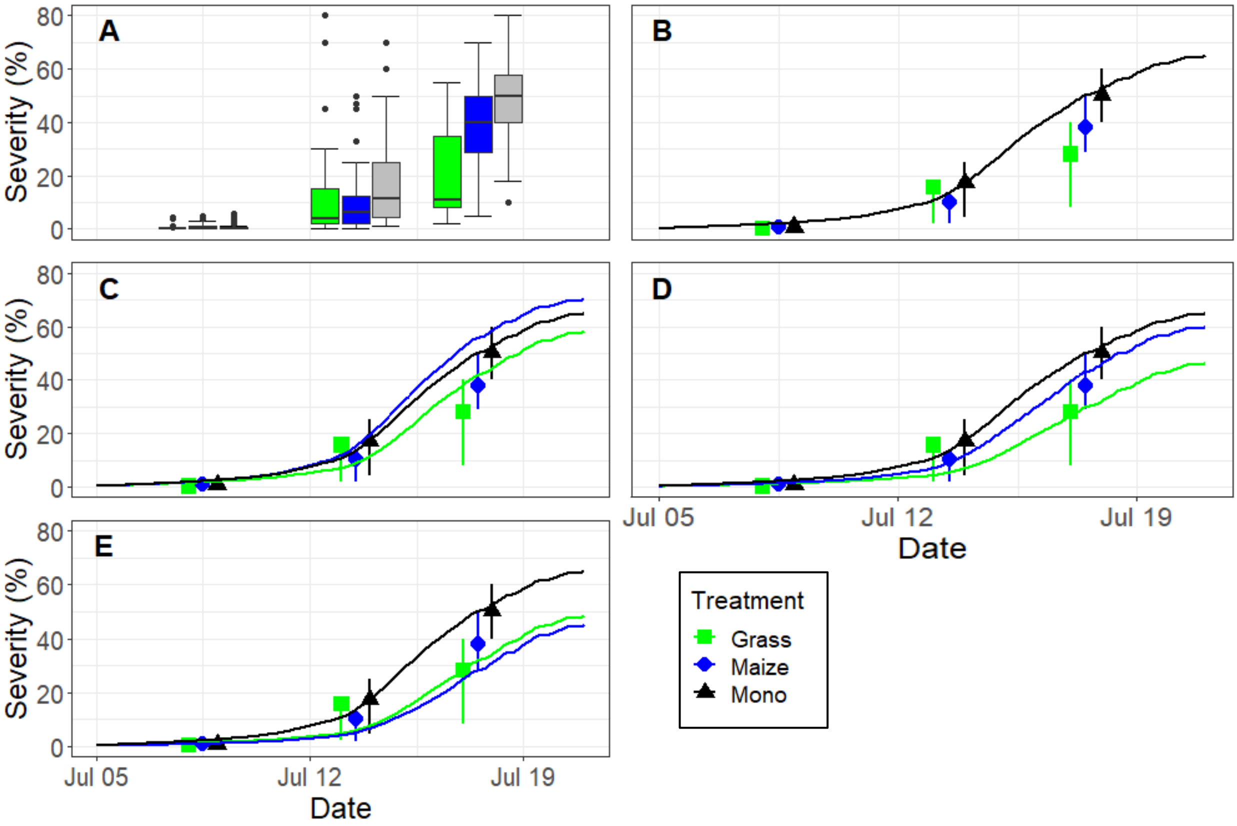

Fig. S8 Validation of late blight simulation model for strip-cropping treatments of 2021. (A) Boxplots of observed plant-level disease severity are depicted. Field observations took place on three dates, July 8, 13 and 17 2021. (B-E) Mean simulated DPC are shown for monoculture and two strip-crop treatments (potato strip-cropped with maize or ryegrass) under various scenarios. Trials of potato strip-cropped with faba bean were not conducted in 2021. Plotted points represent the mean observed disease severity, assessed per plant, and error bars represent upper and lower quartiles. The simulated scenarios that are depicted are: (B) monoculture (C) with the effect of microclimate modification ('MM'), (D) with the combined effects of host dilution and microclimate modification ('HD + MM'), and (E) with the combined effects of host dilution, microclimate modification, and the barrier effect ('HD + MM + B'). Mechanism abbreviations are: HD (host dilution); MC (microclimate modification); B (barrier effect); IR (induced resistance).

References

Arneson, B. E., Ticknor, P. A., Donovan, S., Everse, S., Fass, M., Waterman, M., Stanley, E. D., Cadwallader St Mary, J., Louis, E., & Wilson, R. (1993). *LATEBLIGHT A Plant Disease Management Simulation Version 3.1 User's Manual Editorial Board*.

Crosier, W. (1934). *Studies in the Biology of Phytophthora Infestans (Mont.) de Bary*. Cornell University. Studies in the Biology of Phytophthora Infestans

Fry, & Bruhn. (1980). *Analysis of Potato Late Blight Epidemiology by Simulation Modeling*.

Fry, & Paysour. (1983). *Interplot Interference: A Model for Planning Field Experiments with Aerially Disseminated Pathogens*.

Hartill, W. F. T., Young, K., Allan, D. J., & Henshall, W. R. (1990). Effects of temperature and leaf wetness on the potato late blight. *New Zealand Journal of Crop and Horticultural Science*, *18*(4), 181–184. https://doi.org/10.1080/01140671.1990.10428093

Hjelkrem, A. G. R., Eikemo, H., Le, V. H., Hermansen, A., & Nærstad, R. (2021). A process-based model to forecast risk of potato late blight in Norway (The Nærstad model): model development, sensitivity analysis and Bayesian calibration. *Ecological Modelling*, *450*. https://doi.org/10.1016/j.ecolmodel.2021.109565

MATLAB. (2023). *Version 9.14.0.2239454 (R2023a)*. The Mathworks Inc.

Narouei-Khandan, H. A., Shakya, S. K., Garrett, K. A., Goss, E. M., Dufault, N. S., Andrade-Piedra, J. L., Asseng, S., Wallach, D., & van Bruggen, A. H. C. (2020a). BLIGHTSIM: A new potato late blight model simulating the response of phytophthora infestans to diurnal temperature and humidity fluctuations in relation to climate change. *Pathogens*, *9*(8), 1–20. https://doi.org/10.3390/pathogens9080659

Narouei-Khandan, H. A., Shakya, S. K., Garrett, K. A., Goss, E. M., Dufault, N. S., Andrade-Piedra, J. L., Asseng, S., Wallach, D., & van Bruggen, A. H. C. (2020b). (S.I.)

BLIGHTSIM: A new potato late blight model simulating the response of phytophthora infestans to diurnal temperature and humidity fluctuations in relation to climate change. *Pathogens*, *9*(8), 1–20. https://doi.org/10.3390/pathogens9080659

Rotem, J., Cohen, Y., & Putter, J. (1970). Relativity of Limiting and Optimum Inoculum Loads, Wetting Durations, and Temperatures for Infection by Phytophthora infestans. *Phytopathology*, *61*, 275–278.

Skelsey, P., Kessel, G. J. T., Rossing, W. A. H., & Van Der Werf, W. (2009). Parameterization and evaluation of a spatiotemporal model of the potato late blight pathosystem. *Phytopathology*, *99*(3), 290–300. https://doi.org/10.1094/PHYTO-99-3-0290

Skelsey, P., Rossing, W. A. H., Kessel, G. J. T., Powell, J., & Van Der Werf, W. (2005). Influence of host diversity on development of epidemics: An evaluation and elaboration of mixture theory. *Phytopathology*, *95*(4), 328–338. https://doi.org/10.1094/PHYTO-95-0328

Wallin, J. R., & Hoyman, W. M. G. (1958). Influence of Post-Inoculum Air Temperature Maxima on Survival of Phytophthora infestans in Potato Leaves. *American Potato Journal*, *35*, 769–773. https://doi.org/10.1007/BF02911242

Zwankhuizen, M. J., & Zadoks, J. C. (2002). Phytophthora infestans's 10-year truce with Holland: A long-term analysis of potato late-blight epidemics in the Netherlands. *Plant Pathology*, *51*(4), 413–423. https://doi.org/10.1046/j.1365-3059.2002.00738.x
